# Therapeutic Writing Interventions for Adults With Chronic Pain: Experiences and Health Effects—A Systematic Review With a Narrative Synthesis

**DOI:** 10.1002/ejp.70235

**Published:** 2026-02-16

**Authors:** Toni Salikka, Jari Kylmä, Sari Fröjd

**Affiliations:** ^1^ Social Psychiatry, Health Sciences Unit, Faculty of Social Sciences Tampere University Tampere Finland; ^2^ Nursing Science, Health Sciences Unit, Faculty of Social Sciences Tampere University Tampere Finland

## Abstract

**Background and Objective:**

Chronic pain is often associated with heightened psychological stress, which may exacerbate symptoms and impair functioning. Therapeutic writing has been proposed as a low‐cost adjunctive strategy. This review evaluated the reported experiences and health effects of writing interventions in adults with non‐malignant chronic pain.

**Databases and Data Treatment:**

A systematic search of Web of Science, EBSCOhost, ProQuest, Ovid and Scopus (1999–November 5, 2025) identified studies examining health effects and experiences of therapeutic writing. Methodological quality was assessed using Joanna Briggs Institute tools, and certainty of evidence using GRADE and CERQual.

**Results:**

Twenty‐four studies involving 2477 adults were included. Therapeutic writing supported pain management through emotional processing, cognitive reappraisal and meaning‐making. Health effects were modest, context‑dependent and typically short‑term, with small reductions in pain and improvements in psychological well‐being but limited physical benefits. Effects varied according to individual characteristics, including negative affect and catastrophizing. Qualitative evidence described increased self‑understanding, empowerment and coping, especially in structured, group‐based or cognitive‐behavioral contexts, suggesting that benefits may partly reflect the broader therapeutic environment. Certainty of evidence ranged from low to moderate for quantitative outcomes and moderate to high for qualitative findings.

**Conclusions:**

Therapeutic writing may offer modest but meaningful psychological benefits for adults with chronic pain, although physical improvements appear fleeting. These findings support its use as a low‐intensity adjunct within multidisciplinary pain management. Future research should employ mechanism‑focused, moderator‑sensitive trials and include process‐level measures to clarify how writing exerts its effects and to identify the individuals most likely to benefit.

**Significance Statement:**

This review clarifies when therapeutic writing may be clinically useful in chronic pain care: as a low‐intensity adjunct that can support coping and psychological adjustment in specific contexts and patient groups. By integrating experiential and quantitative evidence, it highlights the importance of delivery structure, emotional pacing and patient characteristics, and identifies targets for better‐designed interventions that can be safely incorporated into multidisciplinary pain management.

## 1 Introduction

Pain enduring beyond 3 months is defined as chronic pain, and profoundly affects various aspects of individuals' lives. Specifically, it is associated with substantial impairment across biological, psychological and social domains (Cohen et al. 2021; Breivik et al. 2006). Those experiencing chronic pain face an elevated likelihood of comorbid mental health issues (Burke et al. 2015; Velly and Mohit 2018). The most prevalent psychiatric disorders that often co‐occur with chronic pain are mood, anxiety, personality, sleep and substance use disorders (Gjerde et al. 2023; Hooten 2016). Additionally, chronic pain has been recognised as a remarkable factor contributing to both diminished quality of life (Inoue et al. 2015; Pandelani et al. 2023) and an increased risk of suicidal behaviour (Themelis et al. 2023).

Epidemiological data underscores the magnitude of chronic pain's impact, affecting an estimated 19% of adults in Europe (Breivik et al. 2006) and 20.9% of adults in the United States (Rikard et al. 2023). An inadequate treatment of chronic pain places a considerable burden on individuals and healthcare services (Breivik et al. 2013), highlighting the urgency of finding alternative solutions for chronic pain management. Therapeutic writing interventions, which involve the practice of writing to promote healing and well‐being, have emerged as a potential tool for chronic pain management. They address the intricate nature of chronic pain by reckoning with cognitive, emotional and behavioural elements that influence how individuals perceive pain and cope with it (Lumley, Cohen, et al. 2011). A growing literature has highlighted the role of cognitive and emotional processing in chronic pain management (Lumley and Schubiner 2019; Williams et al. 2020), suggesting that therapeutic writing could be a valuable complementary approach in this context.

However, the extent to which therapeutic writing interventions impact health outcomes in adults with chronic pain has yet to be fully elucidated. While earlier reviews (Frisina et al. 2005; Lumley et al. 2012) have contributed to the literature, they have predominantly focused on written emotional disclosure, leaving much unexplored. The existing literature on various therapeutic writing for chronic pain is fragmented, with studies varying widely in their methodologies, participant characteristics and outcomes. This variability makes it challenging to draw definite conclusions about the efficacy of these interventions. Moreover, while some studies have reported significant positive effects (Trompetter et al. 2015), others have found minimal (Lumley, Leisen, et al. 2011) or no benefits (Broderick et al. 2004), further complicating the understanding of therapeutic writing on health outcomes for this population.

The goal of this systematic review is to address these gaps by analysing and synthesising published evidence on therapeutic writing interventions for adults with non‐malignant chronic pain (henceforth chronic pain). The primary objectives are to evaluate adults' experiences and the health effects of these interventions. Drawing from randomised controlled trials (RCTs), qualitative studies and a case series, this review seeks to provide a comprehensive understanding of the experiences and health effects of the therapeutic writing interventions. It further aims to inform clinical practice and guide the development of evidence‐based therapeutic writing interventions for chronic pain management.

## 2 Methods

### 2.1 Eligibility Criteria and Search Strategy

This systematic review employed the standards of the Preferred Reporting Items for Systematic reviews and Meta‐Analyses (PRISMA) guidelines (Liberati et al. 2009). The registration of this review's protocol can be found in PROSPERO (CRD42024501332). A university librarian was consulted on the search process. Web of Science, EBSCOhost, ProQuest, Ovid and Scopus databases were searched for the terms ‘chronic pain’ AND ‘writing’ to identify all relevant studies that were available. The search strategy included the use of Boolean operators, truncations and specific field searches (e.g., title, abstract). Filters applied included English‐language studies published between January 1, 1999 and May 24, 2025, because therapeutic writing began to be utilised in chronic pain management starting around 1999 (see, for example, Smyth et al. 1999). Choosing 1999 as the starting point ensures that the review captures the initial emergence and subsequent development of therapeutic writing as a recognised complimentary treatment method for chronic pain. The initial search was conducted October 31, 2023. Updated searches were conducted on May 24 and November 05, 2025. These yielded five additional records, of which two met the inclusion criteria. Additional studies were identified through reference list screening (see Figure S1).

### 2.2 Study Selection

The main author (T. S.) conducted the initial search, carefully reviewing and identifying relevant studies. Following this, the results were thoroughly verified and cross‐checked in collaboration with two co‐authors (J. K. and S. F.) to ensure accuracy and consistency. Each study was then analysed for selection in two stages. In the first stage, the main author (T. S.) initially screened titles and abstracts of the found studies based on predefined exclusion criteria. Studies that did not meet the inclusion criteria were excluded at this stage. In the second stage, the full texts of the remaining studies were retrieved for a detailed review. All authors (T. S., J. K., and S. F.) independently assessed these studies against the inclusion criteria. They evaluated each study for its relevance to the research question, methodological quality, and completeness of data and included those that fully met all the inclusion criteria. Any discrepancies in the assessment were resolved by engaging in discussions and achieving a collective agreement among the authors.

### 2.3 Inclusion and Exclusion Criteria

Inclusion criteria encompassed quantitative, qualitative, or mixed method peer‐reviewed studies and original articles that focused on examining the health effects or experiences of adults living with non‐malignant chronic pain. Chronicity was established either by (a) explicit reporting of pain duration of ≥ 3 months or (b) enrollment based on diagnoses that conventionally denote chronic pain (e.g., rheumatoid arthritis, fibromyalgia, systemic lupus erythematosus), even when duration was not explicitly stated. Eligible studies had to include a writing‐based intervention as a primary or integrated component. Studies including participants with malignant chronic pain were excluded because the nature, treatment methods and aims of managing malignant chronic pain differ significantly from those of non‐malignant chronic pain. Expert opinion texts (Evans and Glover 2012; Hovey et al. 2018), verbal emotional disclosure research (Keefe et al. 2008; Van Middendorp et al. 2009) and studies that presented information about writing interventions among adults who experienced pain not explicitly determined to meet the criteria for chronic pain (Koopman et al. 2005; Kraft et al. 2008; D'Souza et al. 2008; You et al. 2014), and a study including participants with malignant chronic pain (Ressler et al. 2012) were excluded (see Table S1).

### 2.4 Data Extraction

The main author (T. S.) extracted data including study design, participant characteristics, pain and mental health features, intervention details and quality assessment information, and manually recorded it in a characteristics table (see Table S2). If data were missing or unclear, study authors were not contacted. In such cases, the item was recorded as ‘not reported’ and this was noted in the data extraction table. The decision, which data was extracted from the included studies was guided by the review question or objective as suggested by Popay et al. (2006). This approach ensures that the data collected is directly relevant to the research objective, enhancing the validity and focus of the systematic review. To ensure accuracy, the extracted data was independently verified by two co‐authors (J. K. and S. F.). Any discrepancies were addressed and resolved through discussions until a mutual agreement among the authors was reached.

### 2.5 Methodological Quality

All authors (T. S., J. K., and S. F.) independently used The Joanna Briggs Institute Critical Appraisal Tools to assess the methodological quality of the included studies (Lockwood et al. 2015; Barker et al. 2023; Munn et al. 2020). Discrepancies were addressed and solved through collective article review and discussion. The critical assessment of study quality yielded the subsequent ratings: qualitative studies received a score of 10 out of 10, randomised controlled trials achieved scores ranging from 12 to 13 out of 13, and the case series study was scored 10 out of 10. If the answer to the quality enquiry was ‘yes’, each criterion statement received a point, while no points were given for ‘no’ or ‘unclear’ responses.

To complement the appraisal of methodological quality, confidence in the review findings was assessed using the GRADE (Grading of Recommendations Assessment, Development and Evaluation) framework (Guyatt et al. 2008) for quantitative evidence and the GRADE‐CERQual (Confidence in the Evidence from Reviews of Qualitative Research) approach (Lewin et al. 2018) for qualitative findings. These frameworks facilitated a structured evaluation of the certainty and trustworthiness of the synthesised evidence, taking into account factors such as study limitations, consistency, directness, precision and publication bias (GRADE), as well as methodological limitations, coherence, adequacy of data and relevance (GRADE‐CERQual). This dual approach ensured a transparent and rigorous assessment of the overall strength of the evidence base.

### 2.6 Narrative Data Synthesis Approach

As suggested by the literature (Baumeister 2013; Siddaway et al. 2019), a narrative synthesis of data was chosen over meta‐analysis due to the diverse methodologies and different theoretical conceptualizations. More specifically, the significant heterogeneity among the included studies in terms of design, setting, interventions and outcome measures made narrative synthesis a more suitable approach for integrating diverse results (Baumeister 2013). Narrative synthesis allowed for flexible and comprehensive integration and interpretation of diverse findings (Baumeister and Leary 1997), providing a deeper contextual understanding of the experienced health effects of writing interventions.

The narrative synthesis was carried out in accordance with the Guidance on the Conduct of Narrative Synthesis in Systematic Reviews (Popay et al. 2006). This approach involved systematically summarising and explaining the patterns and themes that emerged from the data, considering the context and quality of each study. By doing so, this study aimed to provide a coherent and nuanced interpretation of the evidence, underscoring both the limitations and strengths of the included studies. Although no formal statistical methods were used to explore heterogeneity, potential sources of variation were examined narratively. Studies were grouped by intervention type and outcome domain, and differences in findings were interpreted in light of variations in population characteristics, writing paradigms, and techniques. Given that this review included qualitative studies and studies with diverse reporting formats, many did not provide summary statistics or effect estimates suitable for tabulation. Due to the narrative synthesis approach, no meta‐analysis was conducted.

## 3 Results

A total of 784 studies were identified through database searches and other sources. These studies were evaluated for eligibility adhering to inclusion and exclusion criteria. After the evaluation, 24 studies were included in the final synthesis. The process of selecting studies is depicted in Figure S1.

To support a coherent synthesis of diverse findings, the Section 3 is organised into five subsections: Study Characteristics, Therapeutic Writing Paradigms, Methodological Quality, Lived Experiences and Health‐Related Outcomes, Specifically Pain, Mental Health and Physical Health. This structure enables meaningful comparisons across intervention types and outcome domains while integrating both quantitative and qualitative evidence.

### 3.1 Study Characteristics

The included 24 studies comprised 16 randomised controlled trials, one case series study and seven qualitative studies, with a total population of 2477 adults. The included studies varied in design, population and interventions. Key characteristics are summarised in Table S2.

Although some studies drew from overlapping participant cohorts, each was included as a distinct study due to differences in methodology, timing and analytical focus. For example, Junghaenel et al. (2008) was a secondary analysis of data from Broderick et al. (2005), and Furnes and Dysvik (2012) and Furnes et al. (2014) involved the same cohort at different time points. Furnes et al. (2015) likely included a purposive subsample from a similar program, though overlap could not be confirmed. Participant numbers were reported per study, and all studies were cited individually throughout the review. These overlaps were explicitly noted and considered during synthesis to avoid overrepresentation of findings from a single intervention context.

Most participants were female, with their percentages ranging from 50% to 100% across the included studies. Male participants constituted between 0% and 42.7% of the sample. Notably, one study (Lou et al. 2022) included a non‐binary participant. Participants' ages ranged from 18 to 85 years, with average ages typically falling between 44 and 60 years (see more details in Table S2).

The studies included participants with various chronic pain conditions, including rheumatoid arthritis, fibromyalgia, systemic lupus erythematosus, and chronic pelvic pain. Nine studies reported mixed or unspecified chronic pain conditions. Two studies did not report details regarding pain type nor diagnosis (Marceau et al. 2007; Baker and Wang 2006). The timing of diagnosis varied, with some participants diagnosed as recently as 3.5 years ago and others having lived with their diagnosis for over a decade. The duration of pain ranged from 3 months to over 20 years, with many participants experiencing chronic pain for more than 8 years.

The included studies reported a range of psychological symptoms among participants with chronic pain. Anxiety was noted in eight studies, while depressive symptoms were the most frequently reported, appearing in 11 studies. Emotional distress was highlighted in two studies, and pain catastrophizing was mentioned in three studies. Additionally, emotions such as anger, sadness and fear were reported in a few studies. These observations underscore the significant emotional and psychological burden experienced by individuals with chronic pain. Although formal psychiatric diagnoses were often not reported (see Table S2), sometimes due to the exclusion of individuals with psychiatric disorders (Smyth et al. 1999; Broderick et al. 2005; Kristjánsdóttir et al. 2013; Trompetter et al. 2015). Still, some studies indicated a significant proportion of participants had been diagnosed with depressive (Graham et al. 2008; Veillette et al. 2019) and anxiety (Veillette et al. 2019) disorders.

### 3.2 Therapeutic Writing Paradigms and Techniques

As summarised in Table S3, a total of six therapeutic writing paradigms and 20 distinct techniques were identified through a comprehensive synthesis of the literature. These paradigms (expressive writing, positive writing, journal writing, Acceptance and Commitment Therapy–based writing, integrative therapeutic writing and narrative writing) were derived by analysing the theoretical foundations, therapeutic objectives, and methodological features of the included interventions. In cases where paradigms were not explicitly named by the original authors, classification was guided by a close examination of the intervention's theoretical orientations, defining characteristics, underlying psychological mechanisms and intended outcomes. Each paradigm represents a conceptual framework for the therapeutic use of writing, while the techniques within each paradigm reflect specific implementations or adaptations observed across studies. This categorisation enabled a structured synthesis of diverse interventions and facilitated meaningful comparisons across studies with varying theoretical orientations.

#### 3.2.1 Expressive Writing Paradigm

The expressive writing was the most frequently represented paradigm. It involves writing about emotionally significant or traumatic experiences to promote emotional processing, insight, psychological adjustment and with the aim of reducing pain. The standard written emotional disclosure protocol, originating in the study of Pennebaker and Beall (1986), was utilised in five studies (Broderick et al. 2004; Lumley, Leisen, et al. 2011; Wetherell et al. 2005; Byrne‐Davis et al. 2006; Smyth et al. 1999), where participants engaged in emotional disclosure writing about traumatic or stressful experiences of their past.

Seven studies implemented variations of written emotional disclosure, adapting the protocol to specific contexts. Danoff‐Burg et al. (2006) focused on writing about rheumatic disease, while Gillis et al. (2006) addressed stressful experiences affecting illness and social relationships. Broderick et al. (2005) and Junghaenel et al. (2008) guided participants to reappraise major stressful events, whereas Lumley et al. (2014) investigated a guided writing focused on stressful or traumatic events to promote emotional expression, meaning‐making and coping. Norman et al. (2004) explored negatively valenced emotional experiences related to chronic pelvic pain, and Trompetter et al. (2015) included psychoeducation in addition to emotion‐focused writing assignments related to pain or life stressors. Additionally, two studies (Graham et al. 2008; Van Middendorp et al. 2010) examined written anger expression, with Graham et al. (2008) directing anger toward specific individuals or situations and Van Middendorp et al. (2010) focusing on articulating anger‐related thoughts and feelings.

#### 3.2.2 Positive Writing Paradigm

The positive writing paradigm emphasises writing about uplifting or constructive topics to foster psychological well‐being and resilience. Three studies applied techniques under this paradigm. Broderick et al. (2004) tested written enhanced meaning to reinterpret past trauma, while Danoff‐Burg et al. (2006) implemented written benefit finding related to illness experiences. Ziemer et al. (2015) guided participants to write from a self‐compassionate and self‐efficacy perspective regarding chronic pain. These interventions aimed to cultivate adaptive coping, emotional growth and positive affect.

#### 3.2.3 Journal Writing Paradigm

The journal writing paradigm involves regular, open‐ended writing about daily experiences, emotions and behaviours, typically without specific prompts. This approach supports self‐reflection, emotional regulation and personal insight. Two studies adopted this paradigm. Kristjánsdóttir et al. (2013) used a smartphone‐based daily diary with therapist feedback, while Marceau et al. (2007) employed electronic and paper diaries to track pain, mood, activity and medication use. Both interventions promoted real‐time awareness and behavioural monitoring.

#### 3.2.4 Acceptance and Commitment Therapy–Based Writing Paradigm

This paradigm integrates the six core processes of Acceptance and Commitment Therapy (ACT) including acceptance, cognitive defusion, present‐moment awareness, self‐as‐context, values and committed action as outlined by Hayes et al. (2004). ACT‐based writing aims to enhance psychological flexibility and well‐being. One study (Veillette et al. 2019) implemented self‐administered writing interventions grounded in ACT principles. Participants engaged in structured exercises to reflect on pain‐related experiences through the lens of ACT, promoting emotional openness and value‐driven behaviour.

#### 3.2.5 Integrative Therapeutic Writing Paradigm

Integrative Therapeutic Writing combines elements from expressive, cognitive‐behavioural, and narrative approaches to address both emotional and cognitive dimensions of well‐being. Three studies (Furnes and Dysvik 2012; Furnes et al. 2014; Furnes et al. 2015) exemplified this paradigm. Participants engaged in structured writing tasks focused on expressing and processing life stressors, exploring pain‐related challenges and contributing factors, identifying coping strategies, addressing self‐esteem issues and reflecting on social networks. The interventions aimed to facilitate emotional processing and cognitive restructuring.

#### 3.2.6 Narrative Writing Paradigm

The narrative writing paradigm centres on constructing coherent personal stories to explore identity, meaning and transformation. Grounded in narrative therapy principles (White and Epston 1990), this approach encourages storytelling as a therapeutic tool. Three studies employed distinct narrative techniques. Baker and Wang (2006) used photovoice, combining photographs with written descriptions of pain experiences. Lou et al. (2022) implemented visual art diaries, where participants created drawings and accompanying narratives. Brown et al. (2010) utilised free‐form narrative writing to explore chronic pain, coping and suffering. Although not always explicitly labelled as narrative writing, these interventions aligned with the core principles of narrative therapy.

#### 3.2.7 Cross‐Paradigm Synthesis

Across paradigms, interventions varied in their emphasis on emotional expression, cognitive restructuring, psychological flexibility, and meaning‐making. Expressive and ACT‐based paradigms were the most frequently studied and demonstrated the clearest links to emotional processing and sustained coping. Narrative and integrative approaches often incorporated group‐based or creative formats, suggesting a broader psychosocial orientation. This diversity underscores the adaptability of writing as a therapeutic tool and highlights the importance of tailoring interventions to individual needs, psychological profiles, and contextual factors.

### 3.3 Quality Assessment

The methodological quality of the included studies varied considerably, with notable limitations across both quantitative and qualitative designs. Methodological limitations, such as true randomisation, allocation concealment, blinding of participants and treatment givers, reliability of measurement methods and follow‐up incompleteness, were common across the evidence base of RCTs in the review. These limitations introduce potential bias and reduce confidence in the internal validity of reported effects. Qualitative studies often lacked reflexivity, with few providing clear statements about the researcher's cultural or theoretical backgrounds. The reciprocal influence between the researcher and research was not distinctly discussed. These omissions limit transparency and may affect the interpretive depth of findings.

The JBI critical appraisal of the quality assessment of the included studies revealed a diverse range of methodological rigour. Quantitative studies exhibited variability, with quality scores ranging from 3/13 to 12/13, reflecting a mix of high, moderate and low‐quality studies. The mean quality score for quantitative studies was 7.5, indicating moderate overall quality. Qualitative studies generally showed higher methodological quality, with scores ranging from 6/10 to 10/10 and a mean score of 8.13. These findings highlight the variability in study quality, with qualitative studies typically exhibiting more robust methodologies compared to quantitative studies. Detailed quality assessment scores are provided in Table S2.

To assess the certainty of evidence, the GRADE framework was applied to quantitative findings and the GRADE‐CERQual approach to qualitative findings. Confidence in quantitative outcomes ranged from low to moderate, depending on the strength, consistency and precision of the evidence. For qualitative findings, moderate to high confidence was assigned in most cases, though some themes were downgraded due to limited data adequacy or unclear analytic transparency.

Overall, the quality assessment highlights considerable heterogeneity in methodological rigour across the evidence base. While qualitative studies tended to be more methodologically robust, variability in design quality and reporting standards was observed across all study types. Detailed appraisal scores and GRADE/CERQual ratings are provided in Tables S2 and S4–S7.

### 3.4 Lived Experiences of Adults With Chronic Pain in Writing Interventions

The seven qualitative studies provided rich qualitative insights into the lived experiences of adults with chronic pain who engaged in therapeutic writing interventions. The thematic analysis of the seven qualitative studies revealed a nuanced understanding of how therapeutic writing interventions impact individuals with chronic pain. A narrative synthesis of the findings identified six overarching themes: (1) Making the Invisible Visible, (2) Emotional Catharsis, Cognitive Reappraisal and Meaning‐Making, (3) Enhanced Self‐Awareness and Identity Reconstruction, (4) Connection, Validation and Shared Understanding, (5) Therapeutic Empowerment and Self‐Management and (6) Challenges and Considerations (see Table S4). These themes underscore the multifaceted psychological and emotional benefits of therapeutic writing modalities in chronic pain contexts while also acknowledging the limitations and complexities inherent in such interventions.

#### 3.4.1 Making the Invisible Visible: Expressing the Inexpressible

Participants frequently described chronic pain as an invisible, misunderstood and isolating experience. Writing and multimodal creative techniques, such as photovoice and visual art diaries, enabled individuals to externalise their pain in tangible, communicable forms. Baker and Wang (2006) found that older adults used photovoice to visually capture and narrate the impact of chronic pain, facilitating interpersonal validation and social recognition. Similarly, Lou et al. (2022) reported that visual art diaries allowed participants to depict aspects of pain that were difficult to verbalise, enhancing communication with healthcare providers and family members. Brown et al. (2010) observed that free‐form narrative writing helped participants ‘write’ their pain into existence, fostering a sense of legitimacy and acknowledgment of their suffering. These interventions made the subjective experience of chronic pain more visible and accessible, both to others and to the participants themselves.

#### 3.4.2 Emotional Catharsis, Cognitive Reappraisal and Meaning‐Making

Therapeutic writing interventions consistently facilitated emotional catharsis, meaning‐making and cognitive reappraisal among adults living with chronic pain. Across diverse modalities including written emotional disclosure, free‐form narrative writing, visual art diaries and photovoice participants reported emotional relief, strengthened coping strategies and enhanced psychological insight (Byrne‐Davis et al. 2006; Brown et al. 2010; Lou et al. 2022; Baker and Wang 2006). Writing enabled individuals to externalise and reframe distressing experiences, allowing for the reinterpretation of pain from alternative perspectives and fostering a deeper understanding of their condition (Furnes and Dysvik 2012; Furnes et al. 2014, 2015). This process aligns with cognitive reappraisal, defined as the modification of one's thoughts to alter emotional impact (Gross 2014), and was particularly evident in interventions embedded within cognitive‐behavioural frameworks. Participants described the writing process as both liberating and challenging, with some experiencing emotional strain while others found it empowering and transformative (Furnes and Dysvik 2012; Furnes et al. 2015). Although some experienced effects appeared short‐lived (Brown et al. 2010), the overall findings suggest that therapeutic writing can promote emotional release, facilitate the construction of coherent personal narratives and support adaptive coping through cognitive and emotional restructuring.

#### 3.4.3 Enhanced Self‐Awareness and Identity Reconstruction

Three writing interventions promoted introspection and identity work, helping participants re‐evaluate their relationship with chronic pain. Brown et al. (2010) found that narrative therapy supported the re‐authoring of personal stories, enabling participants to decenter pain from their core identity. Furnes et al. (2015) described how participants employed transition strategies to redefine their self‐concept, fostering resilience and psychological continuity. Lou et al. (2022) reported that visual art diaries encouraged participants to reconsider their limitations, promoting self‐compassion and acceptance. These findings indicate that integrative therapeutic writing and some forms of narrative writing can facilitate identity reconstruction and support the development of a more empowered and integrated self.

#### 3.4.4 Connection, Validation and Shared Understanding

Group‐based and socially contextualised interventions fostered interpersonal connection and mutual validation. Furnes et al. (2014) found that group CBT programs incorporating therapeutic writing created spaces for shared narratives, reducing isolation and enhancing psychosocial well‐being. In Baker and Wang (2006), participants who shared their visual narratives with others reported feeling heard and understood, highlighting the therapeutic value of social recognition. These outcomes underscore the importance of relational contexts in therapeutic writing interventions, where shared understanding can amplify emotional and therapeutic benefits.

#### 3.4.5 Therapeutic Empowerment and Self‐Management

Integrative therapeutic writing and free‐form narrative writing were consistently linked to increased self‐efficacy and improved pain management strategies. Furnes and Dysvik (2012) reported that integrative therapeutic writing within a CBT framework enhanced emotional regulation and coping skills. Subsequent studies by Furnes et al. (2014, 2015) demonstrated that such interventions promoted active coping, reduced pain‐related catastrophizing, and increased perceived control over pain. Brown et al. (2010) similarly found short‐term reductions in catastrophizing and pain intensity following a home‐based narrative writing protocol. These findings suggest that integrative therapeutic writing and narrative writing can serve as a therapeutic tool for fostering autonomy and self‐management in chronic pain populations.

#### 3.4.6 Challenges and Considerations

Despite the reported benefits, several studies reported emotional strain associated with writing interventions. Byrne‐Davis et al. (2006) noted that some participants found written emotional disclosure distressing or fatiguing, a finding that aligns with results from quantitative studies. Some of these studies, which examined written emotional disclosure and its variations, reported an increase in negative mood or affect immediately post‐intervention (Wetherell et al. 2005; Gillis et al. 2006; Broderick et al. 2005; Junghaenel et al. 2008), suggesting that post‐disclosure distress may be a common short‐term response. These outcomes underscore the need for supportive facilitation and individualised pacing. Furnes and Dysvik (2012) and Furnes et al. (2015) similarly observed that while many participants found integrative therapeutic writing liberating and valuable, others struggled with emotional processing and were unable to complete the tasks. Baker and Wang (2006) reported a high attrition rate (51.85%), with participants citing the combination of narrative writing and emotional reflection as particularly demanding. Collectively, these findings highlight the importance of tailoring therapeutic writing interventions to individual readiness and ensuring that emotional support structures are in place.

### 3.5 Health Effects of Therapeutic Writing Interventions

#### 3.5.1 Pain Management and Reduction

Therapeutic writing interventions have demonstrated varying degrees of effectiveness in reducing pain intensity and improving pain‐related functioning. These effects depended on the format of the intervention, the psychological framework employed, and individual participant characteristics. Two major patterns emerged: emotional expression may offer short‐term relief and digital or group formats may enhance accessibility and engagement. The certainty and confidence in the evidence supporting these findings, based on GRADE (for quantitative outcomes) and CERQual (for qualitative syntheses), are summarised in Table S5.

#### 3.5.2 Emotion‐Focused Writing: Expressive and Positive Writing

Expressive writing protocols centred on emotional disclosure have shown modest short‐term reductions in pain intensity across several conditions. For example, Lumley, Leisen, et al. (2011) reported decreased sensory and affective pain at 1‐ and 6‐month follow‐ups in rheumatoid arthritis patients following standard written emotional disclosure. Similarly, Broderick et al. (2005) and Junghaenel et al. (2008) found significant pain improvements at 4 months in fibromyalgia patients who engaged in emotional expression and cognitive reappraisal, though these effects diminished by 10 months. However, Lumley et al. (2014) reported that a variation of written emotional disclosure, an enhanced guided version involving structured prompts about stressful or traumatic events, associated thoughts and feelings, meaning and impact, and coping strategies, was associated with greater pain at 4 and 12 months for patients with rheumatoid arthritis, suggesting that benefits may vary by specific technique and subgroup.

Norman et al. (2004) observed reduced evaluative pain intensity at 2 months in women with chronic pelvic pain who wrote about the emotional impact of their condition, although sensory and affective pain remained unchanged. These findings suggest that emotional expression may facilitate cognitive reappraisal and transient symptom relief, though results are inconsistent and often limited by self‐report measures and lack of active controls.

Positive writing approaches have yielded mixed outcomes. Danoff‐Burg et al. (2006) found reduced pain in patients with systemic lupus erythematosus and high trait anxiety following benefit‐finding writing, while Ziemer et al. (2015) reported improvements in pain severity, acceptance and self‐efficacy after self‐compassionate writing. However, both studies faced methodological limitations, including unclear randomization and incomplete follow‐up.

Anger expression also appears relevant. Van Middendorp et al. (2010) demonstrated that a tendency to inhibit anger (trait anger inhibition) was associated with higher daily pain in women with fibromyalgia, whereas trait anger expression was linked to lower pain when it was matched by actual anger expression in daily life. Similarly, Graham et al. (2008) found that written anger expression improved pain control and reduced pain severity in individuals with heterogeneous pain sources. Together, these findings underscore the importance of individual emotion regulation styles, such as tendencies to inhibit or express anger, in shaping pain experiences and potentially moderating the effects of interventions.

#### 3.5.3 An ACT‐Based Writing Intervention

A writing intervention based on Acceptance and Commitment Therapy principles also showed improvements in pain‐related outcomes. Veillette et al. (2019) found significant improvements in pain‐related disability and acceptance using ACT‐based bibliotherapy, with effects sustained at 3‐month follow‐up. These interventions emphasise psychological flexibility and acceptance, offering a theoretically grounded and scalable approach to chronic pain management. However, the intervention incorporated multiple components such as readings, meditation practices, and therapist contact. It was also compared only to a waitlist control and experienced notable attrition, which limits the strength of conclusions. These factors suggest that the observed effects may reflect the broader therapeutic package rather than writing alone.

#### 3.5.4 Digital and Group‐Based Delivery Formats

Digital and group‐based formats are emerging as promising delivery methods for therapeutic writing. Kristjánsdóttir et al. (2013) demonstrated that a smartphone‐based diary with therapist feedback reduced pain catastrophizing and improved functioning, with effects sustained at 5‐month follow‐up. In contrast, Marceau et al. (2007) found no significant differences in pain outcomes between paper and electronic diaries, though participants preferred the digital format and found it more user‐friendly.

Group‐based interventions incorporating writing, such as integrative therapeutic writing (Furnes and Dysvik 2012; Furnes et al. 2014, 2015), free‐form narrative writing (Brown et al. 2010) and visual arts diary writing (Lou et al. 2022), suggest that writing may enhance emotional insight, coping, and peer support. However, these studies were largely qualitative, lacked controlled comparisons and did not isolate the specific contribution of writing. The absence of follow‐up data further limits conclusions about long‐term effects.

#### 3.5.5 Synthesis of Evidence

Therapeutic writing interventions show potential for supporting pain management, but their effectiveness varies considerably depending on the psychological framework, delivery format and individual characteristics. Written emotional disclosure and its variations may offer modest short‐term relief for some subgroups, though effects are inconsistent and often diminish over time. Positive writing approaches and anger expression techniques suggest that individual emotion regulation styles may moderate outcomes, but methodological limitations reduce confidence in these findings. An ACT‐based writing intervention, while promising, involves multi‐component programs that extend beyond writing alone, making it difficult to isolate the specific contribution of writing. Digital and group‐based formats appear to enhance accessibility and engagement (with limited evidence for superior clinical efficacy), yet the evidence is largely qualitative, lacks rigorous control and often reflects multi‐component interventions rather than writing in isolation. Overall, the certainty of evidence across intervention types remains low to moderate, highlighting the need for more robust, well‐controlled trials to clarify the mechanisms and optimise the design of writing‐based pain interventions.

### 3.6 Mental Health Outcomes

Therapeutic writing interventions impacted a range of mental health outcomes, including mood, anxiety, depression, catastrophizing, and psychological well‐being. The effectiveness of these interventions varied depending on writing type, delivery format and participant‐specific characteristics. A combined GRADE and CERQual assessment of the evidence supporting these mental health outcomes is presented in Table S6.

#### 3.6.1 Written Emotional Disclosure

Three studies examined at‐home written emotional disclosure among predominantly female participants with rheumatoid arthritis. These studies revealed a pattern of short‐term emotional disruption without consistent long‐term psychological benefit. Wetherell et al. (2005) reported a transient increase in negative mood 1 week post‐intervention, with minor improvements at 10 weeks attributed to deterioration in the control group. Similarly, Broderick et al. (2004) found no early psychological benefits or significant long‐term changes, even among adherent participants. Byrne‐Davis et al. (2006) reported perceived benefits, such as catharsis, resolution and improved coping at 10 weeks. Qualitative data from the same study indicated that participants found the writing psychologically meaningful, while some control participants also experienced emotional arousal, suggesting that neutral writing may not function as a true psychological placebo. Overall, written emotional disclosure was associated with initial distress and variable long‐term outcomes. Perceived emotional processing may influence effectiveness, highlighting the importance of considering both quantitative and subjective data.

#### 3.6.2 Variation of Written Emotional Disclosure

Seven studies explored variations of written emotional disclosure among individuals with fibromyalgia, rheumatoid arthritis, systemic lupus erythematosus, and chronic pelvic pain, assessing psychological outcomes. Outcomes were mixed and often transient. Danoff‐Burg et al. (2006) found no significant group effects at a three‐month follow‐up, and Lumley et al. (2014) similarly reported no overall improvement in psychological symptoms compared to control writing across 12 months. Several other studies, including those by Gillis et al. (2006), Broderick et al. (2005), and Junghaenel et al. (2008), reported short‐term increases in negative affect in both at‐home and laboratory settings, reflecting emotional activation during writing. These initial responses typically subsided by three to 4 months, and sustained improvements in psychological well‐being were limited or absent by 10 months. Laboratory‐based variations of written emotional disclosure interventions that emphasised emotional expression and cognitive reappraisal showed more promising results, particularly among participants with higher education or greater interpersonal distress. Trompetter et al. (2015) examined a variation of written emotional disclosure in an internet‐based trial among participants with various chronic pain complaints. The variation of written emotional disclosure group showed short‐term improvements in anxiety, psychological flexibility, mindfulness and emotional life skills, which were largely maintained at 6 months. However, improvements in depressive symptoms were not sustained, and overall effects were modest. Individual characteristics appeared to moderate outcomes. Participants with higher baseline distress, emotional ambivalence or maladaptive coping styles experienced more pronounced psychological gains, as reported by Norman et al. (2004), Broderick et al. (2005) and Junghaenel et al. (2008). Notably, Norman et al. (2004) found that women with chronic pelvic pain who engaged in negatively valenced emotional writing reported increased positive affect, particularly those with high baseline negative affect, compared to those who wrote with a positive emotional focus. Overall, variation of written emotional disclosure interventions elicited modest and variable mental health benefits, with effectiveness shaped by emotional framing, delivery context and participant‐level factors.

#### 3.6.3 Written Anger Expression

In contrast to ‘general’ emotional disclosure, two studies examined the psychological impact of written anger expression. Graham et al. (2008) found that participants who engaged in structured anger‐focused writing over a 9‐week period reported reduced depressed mood and greater perceived control over pain, with meaning‐making partially mediating these effects. The sample included individuals with varying pain sources, many of whom showed clinical or subclinical depression. Complementary findings from a diary study by Van Middendorp et al. (2010) indicated that expressing anger in daily life was associated with lower end‐of‐day pain among women with high trait anger expression. These findings suggest that congruent emotional expression may buffer psychological distress, particularly when aligned with individual emotion regulation styles.

#### 3.6.4 Benefit Finding, Self‐Efficacy and Self‐Compassion Writing

Benefit‐focused and self‐reflective writing interventions revealed modest potential for improving mental health outcomes in individuals with chronic pain. According to two studies, participants reported reductions in depressive symptoms, increased life satisfaction and enhanced positive affect following self‐compassion, self‐efficacy or benefit‐finding writing (Danoff‐Burg et al. 2006; Ziemer et al. 2015). However, these effects were often limited to specific subgroups, such as individuals with high trait anxiety, and did not consistently extend to broader psychological functioning. Moreover, the absence of changes in the targeted constructs, brief intervention durations, reliance on self‐report measures and limited control conditions constrain the interpretability and generalizability of these findings.

#### 3.6.5 ACT‐Based Writing

One self‐administered ACT‐based writing intervention (among other things) showed consistent improvements in mental health among individuals with chronic pain. Veillette et al. (2019) found significant post‐treatment and 3‐month reductions in depression, anxiety and psychological inflexibility, with 54% of participants reporting overall improvement in emotional well‐being. These findings suggest that this ACT‐based writing intervention is associated with small‐to‐moderate improvements across multiple psychological domains and may offer more durable benefits than expressive writing alone.

#### 3.6.6 Integrative Therapeutic Writing

In contrast to symptom‐focused approaches, three qualitative studies embedded in CBT‐informed chronic pain management programs (Furnes and Dysvik 2012; Furnes et al. 2014, 2015) reported enhanced psychological adaptation rather than formal symptom reduction. Participants described greater self‐awareness, cognitive reframing and recognition of pain as a multifaceted experience, which they linked to improved mood regulation and emotional resilience. These benefits were particularly evident in group‐based formats, where shared experiences and peer support facilitated meaning‐making and emotional expression. Expressing difficult thoughts and feelings through writing was perceived as both challenging and transformative, helping individuals reframe their pain narratives and develop more effective coping strategies. When integrated with CBT, therapeutic writing appeared to reduce suffering and support the transition toward better psychological adjustment.

#### 3.6.7 Narrative Writing

Narrative and creative writing approaches also contributed to psychological well‐being by fostering emotional insight and adaptive coping. Brown et al. (2010) employed free‐form narrative writing in a home‐based intervention, where participants described reductions in pain‐related catastrophizing and improvements in emotional processing. The intervention encouraged individuals to articulate their pain experiences in personal and reflective ways, contributing to enhanced coping and psychological insight. Baker and Wang (2006) used photo‐based narrative writing, combining descriptive text with photographs to deepen participants' understanding of their pain and foster a sense of empowerment and awareness. Similarly, Lou et al. (2022) implemented visual arts diary writing, which helped individuals express both emotional and physical aspects of their pain. Participants reported increased motivation to adapt and grow despite their condition. These approaches emphasised personal meaning‐making and values‐driven reflection, suggesting that narrative modalities may support psychological resilience and emotional well‐being.

#### 3.6.8 Synthesis of Evidence

Across writing modalities, psychological outcomes were generally modest and context‐dependent. ACT‐based and integrative approaches showed the most consistent improvements, particularly when interventions emphasised emotional processing, values‐based reflection or peer support. Evidence remains limited, especially for sustained effects. Group formats and structured delivery enhanced engagement and meaning‐making, while individual traits and emotional framing moderated effectiveness, underscoring the need for tailored interventions in chronic pain populations.

### 3.7 Physical Health Outcomes

#### 3.7.1 Fatigue

Fatigue‐related outcomes were reported in a small number of studies, with short‐term improvements observed across variations of written emotional disclosure, particularly those incorporating emotional processing and cognitive restructuring. In participants with systemic lupus erythematosus, both a variation of written emotional disclosure focused on rheumatic disease and written benefit finding targeting positive illness‐related experiences led to reduced fatigue at 3 months (Danoff‐Burg et al. 2006). Similarly, a variation of written emotional disclosure incorporating emotional expression and cognitive reappraisal resulted in decreased fatigue in women with fibromyalgia (Broderick et al. 2005; Junghaenel et al. 2008). However, these improvements were notably short‐lived. In Broderick et al. (2005) and Junghaenel et al. (2008), benefits observed at 4 months had dissipated by the 10‐month follow‐up. Danoff‐Burg et al. (2006) reported reductions at 3 months but did not assess longer‐term outcomes. In the context of chronic conditions, which are marked by persistent and recurring symptoms, transient improvements without evidence of durability carry limited clinical significance. Without long‐term follow‐up, it remains unclear whether these writing interventions foster meaningful or sustained change in fatigue‐related outcomes.

#### 3.7.2 Physical Disability

Six studies evaluated the effects of therapeutic writing interventions on physical disability in chronic pain populations. Findings from four studies using variations of written emotional disclosure (Danoff‐Burg et al. 2006; Gillis et al. 2006; Lumley et al. 2014; Trompetter et al. 2015) reported small to moderate improvements in physical or pain‐related disability at 1 month and approximately 3‐month follow‐up.

Danoff‐Burg et al. (2006) observed these effects among patients with lupus or rheumatoid arthritis, while Gillis et al. (2006) found improvements in fibromyalgia patients, although effects were not evident at 1 month and may have been confounded by symptom worsening in the control group. Norman et al. (2004), also examining expressive writing in women with chronic pelvic pain, found no overall effect on disability but reported improvements in specific subgroups defined by emotional coping styles.

In contrast, one study employing Acceptance and Commitment Therapy–based intervention (Veillette et al. 2019) demonstrated consistent reductions in pain‐related disability. These effects were moderate in size and maintained at follow‐up points ranging from post‐intervention to 3 months. Across studies, physical disability was assessed through self‐report measures; no studies included objective assessments such as clinician ratings or performance‐based tests. The certainty of evidence supporting these physical health outcomes, as evaluated through GRADE and CERQual methodologies, is summarised in Table S7.

## 4 Discussion

### 4.1 Summary of Key Findings

This systematic review synthesised evidence from 24 studies to evaluate how therapeutic writing interventions affect lived experience and health outcomes in adults with non‐malignant chronic pain. Six experiential themes emerged (Table S4): Making the Invisible Visible; Emotional Catharsis, Cognitive Reappraisal, and Meaning‐Making; Enhanced Self‐Awareness and Identity Reconstruction; Connection and Validation; Therapeutic Empowerment and Self‐Management and Challenges and Considerations. Qualitative evidence indicates that writing helps individuals externalise suffering, organise emotional experiences, and foster agency. Quantitatively, health effects were modest, inconsistent and often short‐lived: small, short‐term changes in pain intensity, improvements in coping, acceptance, and psychological flexibility and limited, context‐dependent physical outcomes (Tables S5–S7). Overall certainty was low–moderate (GRADE) for quantitative outcomes and moderate–high (CERQual) for qualitative themes (Tables S4–S6).

### 4.2 Interpretation of Findings and Relationship to Existing Literature

The qualitative findings support a hypothesised progressive account in which pain‐focused therapeutic writing engages an initial phase of affective activation that can be burdensome. When pacing and guidance are adequate, this phase is associated with cognitive reappraisal and meaning‐making (Byrne‐Davis et al. 2006; Furnes et al. 2015). Participants' reports of making pain ‘visible’ through text and visual artefacts lower communicative barriers and enable reinterpretation and narrative organisation, a pattern consistent with expressive‐writing models (Pennebaker and Smyth 2016; Pennebaker and Chung 2011), in which emotional activation is necessary but insufficient; benefit depends on insight, causal integration and coherent storying (Smyth and Pennebaker 2008; Ullrich and Lutgendorf 2002). These mechanisms remain hypotheses rather than demonstrations because most trials did not track linguistic or cognitive markers, limiting mediation and causal inference. The high CERQual confidence for emotional catharsis, cognitive reappraisal, and meaning‐making contrasts with moderate confidence for identity‐related processes, reflecting fewer contributing studies (Baker and Wang 2006; Lou et al. 2022; Furnes et al. 2015) and gaps in reflexivity and ethics (Lou et al. 2022).

A downstream interpretation is that sustained cognitive‐linguistic integration can culminate in identity‐level change and functional self‐management as participants described decentering pain, consolidating more coherent and resilient self‐concepts and experiencing greater agency and perceived control (Brown et al. 2010; Furnes et al. 2015). This trajectory aligns with narrative integration (Pennebaker and Seagal 1999), biographical disruption and repair frameworks (Bury 1982; Charmaz 1995) and adaptation models emphasising meaning‐making and acceptance (Turk and Gatchel 2011). This may partly explain modest improvements in coping (see Table S4) and psychological flexibility in programs embedding writing within CBT contexts (Furnes and Dysvik 2012; Furnes et al. 2014, 2015). However, the identity‐related and second‐theme findings derive from multi‐component interventions, where peer validation, shared problem‐solving (Furnes and Dysvik 2012; Furnes et al. 2014, 2015) and visual art (Baker and Wang 2006; Lou et al. 2022) likely contributed—each a potential active ingredient—limiting attribution to writing alone.

### 4.3 Health Effects of Therapeutic Writing

Across pain, mental health, and physical outcomes, therapeutic writing protocols showed modest, context‐dependent benefits that were generally short‐lived. Written emotional disclosure and its variations yielded short‐term reductions in pain, fatigue or disability in some samples, with psychological benefits most pronounced among individuals with high negative affect, catastrophizing or interpersonal distress. These effects appear moderator‐driven rather than diagnosis‐specific, aligning with meta‐analyses showing small but significant health benefits of expressive writing among individuals high in emotional inhibition or unresolved stress (Frisina et al. 2005; Frattaroli 2006), and with reviews noting modest, inconsistent effects in chronic pain (Lumley et al. 2012).

Benefits typically emerged 1–4 months post‐intervention, whereas immediate distress was common and sometimes persisted for days (Wetherell et al. 2005) or weeks (Gillis et al. 2006). Exposure‐based models view this as evidence that emotional activation is a potential mechanism of therapeutic change (Pennebaker and Chung 2011; Sloan and Marx 2004). Despite that, heightened negative affect alone is not a sufficient predictor of effectiveness (Frattaroli 2006; Sloan et al. 2007). Benefits appear contingent on whether activation is accompanied by cognitive processing, insight and narrative organisation (Pennebaker and Chung 2011). While linguistic indicators of causation, insight, and meaning‐making are more consistently associated with outcomes than raw affect intensity (Pennebaker and Smyth 2016), most expressive writing trials did not assess these processes, leaving mechanisms uncertain.

Beyond disclosure, positively valenced writing showed preliminary, low‐certainty benefits in two small trials (Danoff‐Burg et al. 2006; Ziemer et al. 2015). This echoes non‐clinical findings showing that writing about benefits of adversity (King and Miner 2000) or best possible future selves (King 2001) can improve psychological well‐being and reduce healthcare use. Such evidence suggests that positively focused writing can yield health gains without distressing disclosure (Burton and King 2009; Smith et al. 2018) and be more suitable for individuals with limited tolerance for distress. These findings reinforce prior work that negative emotional focus is not essential for therapeutic benefit (Burton and King 2004; King 2001; Pennebaker and Smyth 2016) and align with recent perspectives integrating personal narratives into positive psychology interventions to enhance agency and resilience in chronic pain (Georgiadis and Johnson 2023), with observed improvements in pain interference, distress and mental health (Müller et al. 2022; Blasco‐Belled et al. 2023; Chakhssi et al. 2018; Pan et al. 2022). Whether benefits arise from valence, cognitive‐linguistic change or their interaction remains uncertain, underscoring the need for trials that include process‐level measures and explicitly test positive writing protocols in chronic pain populations.

### 4.4 Methodological Limitations

This review relied on narrative synthesis, which entails interpretive judgement. Predefined synthesis methods were applied (Popay et al. 2006), and CERQual/GRADE domains were used to enhance transparency (Lewin et al. 2018; Guyatt et al. 2008). Clinical and methodological heterogeneity was substantial, with variability in pain types, intervention formats, and outcome measures, limiting comparability and contributing to inconsistent effects. The search was restricted to English‐language publications, which may have excluded relevant studies and introduced language bias. Data extraction was conducted by a single reviewer, increasing the risk of error despite adherence to a standardised protocol. Study quality varied: few trials reported allocation concealment or blinding, and only five of 16 RCTs achieved complete follow‐up. Most outcomes relied on self‐report, while objective assessments were rare, reducing confidence in physical health findings. Sample characteristics were incompletely reported, and most participants were female, restricting generalizability and obscuring subgroup‐specific effects.

### 4.5 Clinical and Practical Implications

Therapeutic writing appears most promising as a feasible adjunct within multimodal pain management rather than a standalone treatment, aligning with clinical guidelines advocating nonpharmacological, multidisciplinary approaches to chronic pain (Häuser et al. 2021; Dowell et al. 2022). In practice, writing can be:

1. Embedded within CBT or ACT, with prompts targeting appropriate emotional activation, cognitive reappraisal, values and acceptance, supported by pacing and emotional safety.
2. Delivered individually or in groups, depending on the goals and needs.
3. Tailored by emotion‐regulation profile: patients high in negative affect, catastrophizing or interpersonal distress may benefit from expressive writing, whereas those with limited tolerance for increases in negative affect may prefer positive writing that induces positive affect.
4. Balanced in valence by combining positive writing with controlled exposure when indicated to reduce distress while promoting agency and motivation.
5. Supported with safeguards, such as screening for vulnerability, rationales, crisis protocols and optional therapist contact for those with high distress.

### 4.6 Future Research

Future research should move beyond past‐ and present‐tense, negatively valenced writing to explore future‐oriented, goal‐directed writing with positive valence. For some individuals living with chronic pain, an orientation toward future‐directed coping—rather than ruminative immersion in past or present pain‐sensory suffering—may support psychological agency and adaptive functioning by redirecting cognitive and emotional resources from ongoing pain sensations toward meaningful action. This represents a significant and underexplored research frontier. High‐quality trials are needed to:

- Prioritise adequately powered, preregistered trials that directly compare therapeutic writing techniques, doses and delivery formats.
- Use mechanism‐based stratification (e.g., baseline distress, catastrophizing, emotion‐regulation traits, psychological flexibility) and test key mediators (meaning making, reappraisal, acceptance), while employing dismantling, factorial and adaptive designs to isolate active components of writing interventions and personalise dose and content.
- Test moderators across pain phenotypes (nociceptive, neuropathic, nociplastic), psychiatric comorbidities (mood, anxiety, trauma‐related disorders) and sex differences.
- Include multimodal outcomes (patient‐reported, clinician‐rated, performance‐based and biomarkers) and process measures (linguistic analyses of causation, insight, temporal perspective; indices of emotional activation; cognitive reappraisal; psychological flexibility).
- Evaluate durability and maintenance, dose–response relationships and implementation outcomes.
- Ensure ethical safeguards and include diverse cultural and resource settings for external validity.

## 5 Conclusions

Therapeutic writing confers psychological and experiential benefits (emotional processing, identity reconstruction, and empowerment) while quantitative effects on pain, mental health, and physical outcomes remain modest and context dependent. This review consolidates convergent evidence to specify candidate experiential mechanisms and offers practical guidance for targeted adjunctive use in chronic pain care. Its near‐term value is as a low‐intensity adjunct within multimodal care, particularly when integrated with structured guidance and scalable delivery. Realising its potential requires rigorous trials that isolate active components, test mechanisms and moderators, incorporate objective functional measures, and evaluate durability. Until then, clinical use should be targeted, supported and embedded within CBT/ACT‐informed care, with attention to patient selection, valence balance and emotional scaffolding. Clinicians should weigh short‐term distress against potential longer‐term gains and align writing tasks with patient preferences and goals.

## Author Contributions

This systematic review was designed by T.S. The literature search and data extraction were conducted by T.S. The results of the literature search were verified by T.S., J.K. and S.F. The methodological quality of the included studies was critically assessed by T.S., J.K. and S.F. Data analysis was performed by T.S. The manuscript was drafted by T.S., with feedback and improvement suggestions from J.K. and S.F. The final manuscript was reviewed by T.S., J.K. and S.F. and edited by T.S. All authors have approved the final version of the manuscript and agree to be accountable for all aspects of the work.

## Funding

The authors have nothing to report.

## Disclosure

Amendments to Protocol: The PROSPERO registration was refreshed to reflect the completion of all the review stages. The membership of the review team was updated, and the anticipated completion date was extended.

## Conflicts of Interest

The authors declare no conflicts of interest.

## Supporting information


**Data S1:** ejp70235‐sup‐0001‐Supinfo.docx.
