# Supplementary material for: Therapeutic Writing Interventions for Adults With Chronic Pain: Experiences and Health Effects—A Systematic Review With a Narrative Synthesis
Source: Eur J Pain. 2026 Feb 16;30(2):e70235. doi: 10.1002/ejp.70235 (PMC12910190; doi:10.1002/ejp.70235)
Supplement: Supplementary file 1 — Data S1: ejp70235‐sup‐0001‐Supinfo.docx. [file EJP-30-0-s001.docx]

**Supplementary Online Content**

**FigureS1. PRISMA Flow Diagram**

**TableS1. Excluded Studies with Reasons for Exclusion**

**TableS2. Characteristics of Included Studies**

**TableS3. Therapeutic Writing Paradigms and Techniques**

**TableS4. CERQual Summary of Findings: Experienced Health Effects**

**TableS5. Summary of Findings: Pain Management and Reduction (GRADE and CERQual)**

**TableS6. Summary of Findings: Mental Health Outcomes (GRADE and CERQual)**

**TableS7. GRADE Summary of Findings: Physical Health Outcomes**

**FigureS1. PRISMA Flow Diagram**

**Identification of studies via other methods**

**Identification of studies via databases and registers**

Records identified from:

Citation searching (n = 16)

Records removed *before screening*:

Duplicate records removed (n = 21)

Records identified from*:

Databases (n = 768)

Web of Science (n = 237)

EBSCOhost (n = 349)

ProQuest (n = 86)

Ovid (n = 24)

Scopus (n = 72)

**Identification**

Records excluded

(n = 730)

Web of Science (n = 230)

EBSCOhost (n = 336)

ProQuest (n = 81)

Ovid (n = 21)

Scopus (n = 62)

Records screened

(n = 747)

Reports sought for retrieval

(n = 16)

Reports sought for retrieval

(n = 17)

**Screening**

Reports excluded:

Reason 1: Not on chronic pain (n = 1)

Reason 2: Not a writing intervention (n = 2)

Reason 3: Included participants with malignant chronic pain (n = 1)

Reports assessed for eligibility

(n = 16)

Reports assessed for eligibility

(n =17)

Reports excluded:

Reason 1: Ineligible type of reports to be included (n = 2)

Reason 2: Participants did not have chronic pain (n = 3)

Studies included in review

(n =12 + 12 = 24)

**Included**

**TableS1. Excluded Studies with Reasons for Exclusion**

| **Full citation** | **Reason(s) for exclusion** |
| --- | --- |
| Evans, K., & Glover, L. (2012). “Finding the unexpected”: An account of a writing group for women with chronic pelvic pain. *Journal of Poetry Therapy*, *25*, 95–103. https://doi.org/10.1080/08893675.2012.680724 | This is an expert’s opinion text. The report did not utilize a qualitative, quantitative, or mixed methods. Therefore, it failed to meet the inclusion criteria. |
| Hovey, R. B., Khayat, V. C., & Feig, E. (2018). Cathartic Poetry: Healing Through Narrative. *The Permanente Journal*, *22*, 17–196. https://doi.org/10.7812/TPP/17-196 | This is an expert’s opinion text. The report did not utilize a qualitative, quantitative, or mixed methods. Thus, it did not fulfil the inclusion criteria. |
| Keefe, F. J., Anderson, T., Lumley, M., Caldwell, D., Stainbrook, D., Mckee, D., Waters, S. J., Connelly, M., Affleck, G., Pope, M. S., Weiss, M., Riordan, P. A., & Uhlin, B. D. (2008). A randomized, controlled trial of emotional disclosure in rheumatoid arthritis: Can clinician assistance enhance the effects? *Pain*, *137*, 164–172. https://doi.org/10.1016/j.pain.2007.08.031 | Not a writing intervention. The intervention was purely verbal: it was not written emotional disclosure but spoken emotional disclosure. Therefore, the report did not satisfy all inclusion criteria. |
| Van Middendorp, H., Geenen, R., Sorbi, M. J., van Doornen, L. J. P., & Bijlsma, J. W. J. (2009). Health and Physiological Effects of an Emotional Disclosure Intervention Adapted for Application at Home: A Randomized Clinical Trial in Rheumatoid Arthritis. *Psychotherapy and Psychosomatics*, *78*, 145–151. https://doi.org/10.1159/000206868 | Not about a writing intervention. The intervention was purely oral: It was not a written emotional disclosure. It was only a spoken emotional disclosure. Thus, the report did not meet all the inclusion criteria. |
| Koopman, C., Ismailji, T., Holmes, D., Classen, C. C., Palesh, O., & Wales, T. (2005). The Effects of Expressive Writing on Pain, Depression and Posttraumatic Stress Disorder Symptoms in Survivors of Intimate Partner Violence. *Journal of Health Psychology*, *10*, 211–221. https://doi.org/10.1177/1359105305049769 | Participants’ pain was not chronic, insofar as it was not defined that their pain did fill the terms of chronic pain. Consequently, the study did not fulfil the specified criteria for inclusion. |
| Kraft, C. A., Lumley, M. A., D’Souza, P. J., & Dooley, J. A. (2008). Emotional approach coping and self-efficacy moderate the effects of written emotional disclosure and relaxation training for people with migraine headaches. *British Journal of Health Psychology*, *13*, 67–71. https://doi.org/10.1348/135910707X251144 | Participants reported experiencing episodic migraine headaches at least once per month, which does not meet the standard definition of chronic pain (pain occurring on most days for ≥3 months). Therefore, this study does not satisfy the inclusion criteria for chronic pain. |
| D’Souza, P. J., Lumley, M. A., Kraft, C. A., & Dooley, J. A. (2008). Relaxation training and written emotional disclosure for tension or migraine headaches: A randomized, controlled trial. *Annals of Behavioral Medicine*, *36*, 21–32. https://doi.org/10.1007/s12160-008-9046-7 | Participants migraine and tension headaches did not fill the terms of chronic pain, insofar as it was not reported that the participants would have had those type of pains longer than one month. For this reason, the study was deemed ineligible for inclusion. |
| You, D. S., Creech, S. K., Vichaya, E. G., Young, E. E., Smith, J. S., & Meagher, M. W. (2014). Effect of written emotional disclosure on secondary hyperalgesia in women with trauma history. *Psychosomatic Medicine*, *76*, 337–346. https://doi.org/10.1097/PSY.0000000000000064 | Did not meet the inclusion criteria, insofar as the participants did not have real chronic pain, that is, the participants experienced for a short period of a laboratory model of chronic pain that was capsaicin-induced pain. Inclusion criteria were not met. |
| Ressler, P. K., Bradshaw, Y. S., Gualtieri, L., & Chui, K. K. H. (2012). Communicating the experience of chronic pain and illness through blogging. *Journal of Medical Internet Research*, *14, e143*. https://doi.org/10.2196/jmir.2002 | The study was excluded because some participants had certain types of cancer, resulting in malignant chronic pain, which did not meet the inclusion criteria. |

**TableS1** lists studies excluded during full-text screening, along with the primary reason for exclusion based on predefined eligibility criteria.

**TableS2. Characteristics of Included Studies**

| **First Author, Year of Publication, and Country** | **Study Design** | **Participant Characteristics (total number, sex, and age)** | **Pain Diagnosis or Type, Timing of Diagnosis, and Duration of Pain** | **Psychological Symptoms or Psychiatric Diagnoses** | **Study Intervention and Location** | **Comparison** | **Writing Intervention Timing, Intensity, and Frequency** | **Main outcomes / Themes and Findings** | **Main Outcome Assessment Time Points** | **Assessment Methods / Measurement Tools and Techniques)** | **Quality Assessment Scores (Joanna Briggs Institute)** |
| --- | --- | --- | --- | --- | --- | --- | --- | --- | --- | --- | --- |
| Broderick et al., 2005, United States of America. | Quantitative research, randomized controlled trial. | N = 92.  Sex: female 100%.  Average age: 50 (range 48–51) years. | Formally diagnosed with FM for an average of 9 years ago. | Anxiety and depressive symptoms, formal psychiatric diagnoses: NR (a major psychiatric disorder was an exclusion criterion). | VWED (focused on emotional expression and cognitive reappraisal of a major stressful event).  Conducted in a private office within a laboratory setting. | NWRIT about day-to-day activities and usual care condition. | Participants wrote for 20 minutes on three consecutive days. | Significantly decreased fatigue and pain.  Significantly enhanced psychological well-being.  The benefits did not persist at 10-month follow-up. | Baseline,  4 months, and  10 months. | QOL, STAI-S, BDI-II, MPI**,** MPQ, MOS, and FIQ. | 7/13**.** |
| Broderick et al., 2004, United States of America. | Quantitative research, randomized controlled trial. | N = 270.  Sex: female 81 %, male 19 %.  Average age: 57.6 (range: 18–NR) years. | Formally diagnosed with RA, timing of the diagnosis and duration of pain: NR. | NR. | Standard WED and WEM (in which the focus was on enhanced meaning of participants’ past trauma).  At home (79 %) or in some other private place. | TMW about daily activities and EVID about RA. | Participants wrote for 20 minutes on three consecutive days. | Feasibility confirmed with 79% participation and 49% adherence. No significant effects on measured primary outcomes. Emotional and stress responses observed; pretreatment differences complicated results. | Baseline,  4 months, and  6 months. | DAS and SF36v2. | 8/13. |
| Danoff-Burg et al., 2006, United States of America. | Quantitative research, randomized controlled trial. | N = 75.  Sex: female 82.7%, male 17.3%.  Diagnoses: SLE 28% (72%: NR).  Average age: 51.2 (range: 18–NR). | Formally diagnosed with SLE on average of 15 years before study participation. Duration of pain: NR. | Anxiety and depressive symptoms. Psychiatric diagnoses: NR. | VWED (about rheumatic disease) and WBF (positive thoughts and feelings concerning an experience with an illness).  In a laboratory, in a private room. | Disease fact control (related to illness and its treatment, unemotionally). | Over the course of three weeks, participants wrote for 20 minutes on four discrete writing sessions. | Significantly reduced fatigue in WBF and VWED at 3-month follow-up. Reduced pain levels in WBF group for high trait anxiety. No significant effects on psychological functioning or disability. | Baseline, 1 month,  and  3 months. | MHAQ, CES-D, POMS, VAS, STAI (Form Y2). | 8/13. |
| Gillis et al., 2006, United States of America. | Quantitative research,  randomized controlled trial. | N = 83.  Sex: female 97.2% female, male 2.8% male.  Diagnosis: FM.  Average age: 50.3 (range = 23–72) years. | Formally diagnosed with FM with a mean of 5.9 years prior to study entry. Duration of pain: NR. | Negative mood, anxiety and depressive symptoms, psychiatric diagnoses: NR. | VWED (about a stressful experience that keeps bothering in the present and how this has impacted the disease, coping with it, or social relationships).  Conducted at home with provided instructions. | TMW (about how one’s time management reduces stress and improves mood: unemotionally focusing on actual behaviors or planned actions). | Participants engaged in consecutive writing sessions lasting 15 to 20 minutes for four days. | VWED led to an immediate increase in negative mood during writing sessions. Few short-term health benefits at 1-month follow-up. VWED resulted in greater reductions in poor sleep, global impact, physical disability, and utilization of health care services than TMW. | Baseline,  1 month, and  3 months. | PANAS-X, FIQ, FSS, AIMS 2, and SQS. | 10/13. |
| Graham et al., 2008, United States of America. | Quantitative study, randomized controlled trial. | N = 102.  Sex: female 57.3%, male 42.7%.  Diagnoses: NR.  Average age was 46.3 (range = 22–70) years. | Heterogeneous, non-mutually exclusive pain sources: arthritis (22.4%), injury (57.2%), complex regional pain syndrome (9.7%), and other (27.5%). Pain experienced at least 6 months prior to the study. | Sadness, anxiety, and depressive symptoms. Psychiatric diagnoses: 37% of the sample was reported to be clinically depressed. Additionally, 30% of the sample showed indications of mild depression. | WAE (focusing on felt anger toward a person or a thing).  First task: 75% of the participants wrote at home, rest in a private room at a pain center. Second task: all completed at home and returned by mail. | NWRIT (about plans for the upcoming day non-emotionally). | Participants wrote a letter for at least 20 minutes in two distinct sessions, approximately 2.5 weeks apart. | Significant enhancement in control over pain and depressed mood in the WAE group compared to control. Marginally significant reduction in pain severity. Degree of expressed anger accounted for intervention effects. Meaning-making mediated effects on depressed mood. | Baseline,  4 weeks, and  9 weeks. | CES-D, MPI, SOPA, LOT-R, and CAB-V. | 10/13. |
| Junghaenel et al., 2008, United States of America. | Quantitative research, randomized controlled trial. | N = 92.  Sex: female 100% female.  Diagnosis: FM.  Average age: 49.7 (range = NR) years. | Formally diagnosed with FM an average of 4.78 years ago. Years with symptoms an average of 8.68 years. | NR. | VWED (focused on emotional expression and cognitive reappraisal of a major stressful event).  Conducted in a private office within a laboratory setting. | NWRIT (about daily activities in connection with the time they dedicated to tasks. | Timing: NR. Participants completed three 20-minute writing sessions. | Significant improvements in psychological well-being, pain, and fatigue for interpersonally distressed patients.  Greater psychological well-being improvements for patients with higher education.  No significant effects for patients with adaptive or dysfunctional pain-coping styles. | Baseline and  4 months. | MPI. | 3/13. |
| Kristjánsdóttir et al., 2013, Norway. | Quantitative research, randomized controlled trial. | N = 140.  Sex: female100%.  Average age: 44.23 (range 18–NR) years. | Formally diagnosed with FM (80.74%): not reported when. Duration with symptoms an average 14.25 years. | Emotional distress and pain catastrophizing. Psychiatric diagnoses: NR (being diagnosed with a profound psychiatric disorder was an exclusion criterion). | SWI (daily electronic diary, personalized therapist feedback based on cognitive behavioral principles, and self-help pain management material online).  Conducted interactively and remotely via smartphones at home. | Control group (provided with access to a non-interactive self-management website that consisted of self-help pain management materials, post 4-week inpatient rehabilitation). | The intervention group wrote three smartphone diary entries daily for four weeks. Intensity: NR. | Significantly improved pain catastrophizing immediately after the intervention and at 5-month follow-up.  Improved acceptance of pain and functioning, and lower symptom levels in the SWI group compared to the control group at follow-up. | Baseline and  5 months. | PCS, CPAQ, GHQ-12, CPVI, VAS, FIQ, and SF-8. | 9/13. |
| Lumley et al., 2011, United States of America. | Quantitative research, randomized controlled trial. | N = 181.  Sex: female 84% (16%:NR).  Average age: 54.6 (range of 20 to 74) years. | Formally diagnosed with RA 11.2 years ago. Years with symptoms: NR. | Anger, fear, sadness, stress, emotional distress, anxiety and depressive symptoms. Psychiatric diagnoses: NR. | Standard WED (of traumatic or stressful experience).  Conducted at home, in a quiet private place. | Positive writing (related to a positive emotional event) and NWRIT (about daily activities non-emotionally) | Across four consecutive days, the intervention group (writing sample) engaged in daily writing for 20 minutes. | Minimally reduced pain at 1 and 6 months. Improved affective pain and increased walking speed. | Baseline,  1 month, and  6 months. | PANAS-X, SF-MPQ, AIMS2, pain behavior videotape, walking speed and grip strength, swollen joint count, PGA, and ESR. | 9/13. |
| Lumley et al., 2014, United States of America. | Quantitative research, randomized controlled trial. | N = 264.  Sex: female 81.1%, male 18.9%.  Average age: 55.1 (range = 22–82) years. | Formally diagnosed with RA an averaged 13.1 years ago. | Psychological symptoms measured (AIMS2 mood/tension); baseline descriptive data not reported. Major psychiatric disorders were exclusion criteria. | Enhanced guided VWED (with structured prompts: stressful or traumatic events, associated thoughts and feelings, meaning and impact, coping strategies).  Conducted in the laboratory or clinic and partly at home. | NWRIT (that focused on factual descriptions of time use, eating, physical activity, and sleep, avoiding emotions or opinions). | The first 20-minute writing session was completed in clinic; three additional sessions were scheduled at home within one week, with adherence verified by phone and returned writings. | VWED demonstrated mixed results: it reduced disease activity and physical disability at the 1-month follow-up compared to control writing, but was associated with greater pain at 4 and 12 months. | Baseline,  1 month,  4 months, and  12 months. | AIMS2, MPQ, swollen joint count, PGA, walking speed (50-foot timed walk), and  C-reactive protein (CRP). | 10/13 |
| Marceau et al., 2007, United States of America. | Quantitative research, randomized controlled trial. | N = 42.  Sex: female 69 % (31%: NR).  Average age: 48 (range = 34–65) years. | Pain type and formal diagnoses: NR.  On average, the participants had experienced chronic pain for longer than 8 years. | Anxiety and depressive symptoms, and anger. Psychiatric diagnoses: NR. | Electronic diary condition (about pain, activity inference, mood, medication use, and pain location monitoring).  Undertaken at home: (partly) online. | Paper diary writing (about pain, activity inference, mood, medication use, and pain location monitoring). | Participants wrote daily for two weeks, then there was a 1-week break, and it was followed a daily writing for two weeks. Intensity: NR. | Electronic diaries were significantly easier to use and preferred over paper diaries. More frequent provided suggestions for medication changes with electronic diaries. There were no discernible distinctions between paper and electronic tracking regarding mood, medication, pain interference, and pain descriptors. | 2 weeks and  4 weeks. | BPI. | 4/13. |
| Norman et al., 2004, United States of America. | Quantitative research, randomized controlled trial. | N = 48.  Sex: female 100%.  Average age: 38.2 (range = 18–64 years). | Formal diagnosis of CPP. The mean duration of CPP was 12.7 years.  Pain affected participants for a mean of 77.0% of the time within a month. | Among the participants, 75% exhibited depressive symptoms, and 64.6% had received mental health treatment. Formal psychiatric diagnoses: NR. | VWED (emotional experiences attached to CPP with a negative valence).  Performed in a private place. | Positive writing (about positive emotional experiences unrelated to CPP) | Over a span of three days, participants wrote for at least 20 minutes each day. | Decreased evaluative pain intensity ratings and disability in VWED.  Higher baseline negative affect was associated with a positive response to disclosure.  Higher baseline catastrophizing was linked to less disability after disclosure.  Affective and sensory pain, overall affect, and disability did not show significant main effects. | Baseline and  2 months. | AEQ, PANAS-X, MPQ, CSQ, and SIP. | 7/13. |
| Trompetter et al., 2015, Netherlands. | Quantitative research, randomized controlled trial. | N = 238.  Sex: female 76.05% (23.95%: NR).  Average age: 52.78 (range 18–NR) years. | Participants had various chronic pain complaints, but only one diagnosis, FM, was reported. (formality: NR). Duration of pain in months: > 6–NR. | Severe levels of anxiety and depression. Psychiatric diagnoses: NR (candidates with severe psychological distress and those diagnosed with major depressive disorder were excluded). | Guided self-help program grounded in Acceptance and Commitment Therapy (ACT), targeting pain-related outcomes.  Undertaken online. | VWED (including psychoeducation and emotion-focused writing assignments (positive and negative) related to pain or life stressors; supported by a personal online diary.  Waiting list control condition (no interventions were conducted). | The VWED control group was instructed to write a minimum of three times a week for about 15 minutes, over a 9–12-week period. | The ACT group showed superior, clinically significant improvements in pain interference, intensity, and catastrophizing, as well as depression and psychological inflexibility compared to the VWED group, but not compared to the waiting list.  The VWED group showed some improvements, but they were not as significant as those seen in the ACT group. | Baseline,  3 months, and  6 months. | MPI, HADS, NRS, PDI, MHC-SF, PIPS, FFMQ-SF, ELS, PCS. | 8/13. |
| Smyth et al., 1999, United States of America. | Quantitative research, randomized controlled trial. | N = 112.  Sex: female 72.43% (27.57%: NR).  Average age: 45.6 (range 18–NR) years. | Formal diagnosis of (asthma or) RA. Timing of diagnosis: ≥ 1 year prior to study enrollment (exact duration not reported). | Exclusion criteria: current psychotherapy or a diagnosed psychiatric disorder. Psychological symptoms: NR (measured in study but not reported at baseline). | Standard WED (about the most stressful experience that participants had ever personally undergone).   In private laboratory rooms. | NWRIT (about emotionally neutral topics). | Participants wrote for 20 minutes on 3 consecutive days. | Writing about stressful experiences (WED) led to improvements in objective physical health outcomes — reduced rheumatoid arthritis disease activity and pain, and improved asthma symptoms and pulmonary function. Psychological symptoms were not assessed or reported to show significant baseline or long-term changes. | Baseline, 2 weeks, 1 month, and 3 months. | PGA, Forced Expiratory Volume in 1 Second (FEV1) measured by spirometry according to ATS guidelines (American Thoracic Society), and Self-Reported Psychosocial Environment collected via daily diaries 1 week prior to and 2 weeks after the writing intervention. | 12/13 |
| Veillette et al., 2019, Canada. | Quantitative research, randomized controlled trial. | N = 140.  Sex: female 81.50%, male 18.50%.  Average age: 51.06 (range 18–NR) years. | Participants had various formal chronic pain diagnoses. More than 41% of them had experienced chronic pain for more than a decade. | Nearly 18% declared they had a diagnosis of a depressive disorder, 16.9% had an anxiety disorder diagnosis, and 7.7% had multiple psychological diagnoses. | Self-administered ACTW (related to pain) with minimal therapeutic contact, including reading materials and exercises to improve psychological flexibility and pain management).  Writing was conducted at home. | Control group (wait-list condition: did not receive any intervention during the study period). | NR. | The ACT intervention showed statistically significant mitigation in pain-related disability and depression, and enhanced psychological flexibility and pain-related acceptance compared to the control group. | Baseline and  3 months. | Sociodemographic and clinical information questionnaire, BPI, BDI-SF, CPAQ, and PIPS. | 6/12. |
| Wetherell et al., 2005, United Kingdom. | Quantitative research, randomized controlled trial. | N = 34.  Sex: female 82.35%, male 17.65%.  Average age: 60.89 (range: NR) years. | All participants had a formal diagnosis of RA. Mean duration of RA: 14.95 years. Duration of pain: NR. | Tension, anger and depressive symptoms. Psychiatric diagnoses: NR. | Standard WED (about personal traumatic experiences).  Conducted at home. | Control group (wrote or spoke about tasks they had accomplished in the past or planned upcoming tasks in a factual, detailed, and unemotional way). | Participants wrote (n = 7 spoke) for periods of 20 minutes on four consecutive days. | The WED group showed an increase in negative mood and disease activity markers one-week post-intervention.  Over time, there were minor improvements in mood and stability in disease activity for the WED group compared to the control group. These differences were due to deteriorations in the control group. | Baseline,  1 week,  3 weeks,  6 weeks, and 10 weeks. | DAS, ESR, CRP, POMS-SF, Likert Scale, and SACL. | 5/13. |
| Ziemer et al., 2015, United States of America. | Quantitative research, randomized controlled trial. | N = 93.  Sex: 86.0% female (14%: NR).  Average age: 49.6 (range 19–74) years. | Various forms of musculoskeletal chronic pain were reported, specific formal diagnoses: NR. Duration of pain: > 8 years (63.4%). | Depressive symptoms and pain catastrophizing. Psychiatric diagnoses: NR. | Positive writing (about one’s chronic pain experiences from a self-compassionate standpoint).  Online-based writing. | Positive writing (about one’s chronic pain experiences with a focus on self-efficacy). | Participants were asked to write for 20 minutes per week for three weeks in succession. | Significant decreases in average reported pain severity, a significant increase in reported life satisfaction, and a significant main effect for positive affect were observed over the three writing sessions following both writing conditions.  There was no significant change in self-compassion or self-efficacy. | Baseline and  3 weeks. | IIRS, CPAQ-8, SCS, CES-D, SWLS, PANAS, PCS, CPSS, and subjective evaluation of writing tasks. | 4/13. |
| Van Middendorp et al., 2010, Netherlands. | Quantitative research, case series study. | N = 333.  Sex: female 100%.  Average age 47 (range = 18–85) years. | Formally diagnosed with FM: an average for 3.5 years ago. Duration of pain: 11 years. In addition, participants had various comorbid diagnoses. | Psychological symptoms: 57% of the participants exhibited symptoms of depression and post-traumatic stress disorder. Psychiatric diagnoses: NR. | WAE (daily diary about anger-related feelings and thoughts in a detailed and reflective manner).  Not explicitly reported where participants wrote. | Control (N/A). | Participants wrote daily for 28 consecutive days (intensity: NR). | State anger inhibition showed no significant association with pain.  Trait anger inhibition was significantly related to increased pain.  Trait anger expression was significantly associated with decreased pain intensity. | Daily. | SECS, STAXI, a diary assessment, and a single-item pain scale. | 9/10. |
| Baker and Wang, 2006, United States of America. | Qualitative research, action research. | N = 27.  Sex: female 67% (33%: NR).  Average age: 65 (range: NR) years. | Diagnosis and duration of pain: >3 months–NR. | NR. | Photovoice (photographs and narrative written descriptions of experience of chronic pain).  Not explicitly reported where participants wrote. | Control (N/A). | Participants were given two weeks to complete the intervention assignments. Intensity and frequency: NR. | Allowed participants to share their chronic pain experiences with others.  Experienced their participation as a means to assist others managing pain.  Potential impact on healthcare providers, policymakers, and community about the lived experiences of chronic pain. | 2 weeks. | An exit interview survey: that assessed participants’ residential information, demographic characteristics, pain experience, and participation in the study. | 6/10. |
| Brown et al., 2010, Canada. | Qualitative research, narrative therapy. | N = 10.  Sex: female 50% female, 30% male (20%: NR).  Average age: NR. Age range = 28–67 years. | Mixed pain. Diagnosis: NR. Range of years with chronic pain among all the participants was 5–30 years. | Anger and catastrophizing. Psychiatric diagnoses: NR. | NW (about chronic pain experience and coping: nociception, pain, suffering, and behavior).  Writing tasks were performed at home. | Control (N/A). | Participants were instructed to engage in a 20-minute writing once a week for five consecutive weeks. | Emergent themes: control, anger, withdrawal, and activity.  Initial improvements in coping styles (reduced anger and catastrophizing).  Benefits not sustained over 3-month follow-up.  Potential for reducing unhelpful cognitions and behaviors. | Baseline,  1 month, and  3 months. | CSQ, SOPA, PDI, CAS, and narrative writing. | 8/10. |
| Byrne-Davis et al., 2006, United Kingdom. | Qualitative research, thematic analysis. | N = 15.  Sex: female 86.67%, male 13.33%.  Average age is 57.6 (range: NR) years. | Diagnosis: RA (formality and timing of diagnosis: NR). Mean duration of reported RA: 15 years. | NR. | Standard WED (about personally stressful or traumatic experiences).  Conducted at participants’ homes. | NWRIT (regarding past or upcoming tasks unemotionally). | Participants attended four sessions each lasting a minimum of 20 minutes, held on four subsequent days. | Participants experienced catharsis, resolution, and realized they had successfully developed coping mechanisms. | 1 week and 10 weeks. | Semi-structured interviews: that were audiotaped. | 6/10. |
| Furnes and Dysvik, 2012, Norway. | Qualitative research, an exploratory-descriptive approach, incorporating a phenomenological perspective. | N = 34, same sample as Furnes et al. (2014, 2015).  Sex: female 70.59%, male 29.41%.  Average age: 49 (range: NR) years. | Pain diagnosis: NR.  The participants had various non-malignant chronic pain problems, in several body regions.  Duration of pain: > 6 years (majority). | NR. | ITW (about current life situation, pain problem and factors, coping strategies, self-esteem and social network, thinking patterns, and communication).  Group-based pain management programme for outpatients who engaged in integrative therapeutic writing as part of their Cognitive Behavioural Therapy (CBT) homework tasks.  Writing was conducted at home. | Control (N/A). | Participants wrote about five themes during the eight-week program. Intensity and frequency: NR. | Three thematic findings: Enhanced comprehension of chronic pain as a complex experience.  Novel approaches for managing chronic pain-related situation.  Therapeutic writing is experienced in diverse ways, both positively and negatively, depending on the writing performance and the individual's unique pain experience. | 8 weeks. | Data were collected through written reports from integrative therapeutic writing. | 9/10. |
| Furnes et al., 2014, Norway. | Qualitative research, a phenomenological hermeneutic approach. | N = 34 (same participants from Furnes and Dysvik, 2012; submitted written reports 6 months post-program.  Sex: NR.  Average age: 49 (range: NR). | Pain diagnosis: NR. The participants had various non-malignant chronic pain, in multiple body regions.  Duration of pain: > 6 years (61.76%). | NR. | ITW (about current life situation, pain problem and factors, coping strategies, self-esteem and social network, thinking patterns, and communication).  Group-based pain management programme for outpatients who engaged in integrative therapeutic writing as part of their Cognitive Behavioural Therapy (CBT) homework tasks.  Writing was conducted at home. | Control (N/A). | Participants wrote about five themes during the eight-week program. Intensity and frequency: NR. | Main Theme: Successful self-management linked to group contributions. Subthemes: Active involvement enhanced insights into pain management; Shared experiences and group support were significant. | 8 weeks. | Data were gathered through written reports that included open-ended questions about group participation and self-help accomplishments. | 10/10. |
| Furnes et al., 2015, Norway. | Qualitative research, an exploratory-descriptive approach, incorporating a phenomenological perspective. | N = 12, drawn from similar CBT program; likely a purposive subsample, not confirmed to be same individuals as in Furnes and Dysvik, 2012 and Furnes et al., 2014.  Sex: female 75.0%, male 25.0%.  Average age: 52 (range: NR) years. | Pain diagnoses: NR.  Chronic pain conditions related to musculoskeletal disorders, neuropathic pain, and late effects of previous oncological treatment (indicating past oncological treatment). Duration of pain: > 10 years (70%). | Depressive symptoms. Psychiatric diagnoses: NR. | ITW (about current life situation, pain problem and factors, coping strategies, self-esteem and social network, thinking patterns, and communication).  Homework writing tasks were accomplished at home. | Control (N/A). | Participants wrote about five themes during the eight-week program. Intensity and frequency: NR. | Three thematic findings: Suffering from chronic pain involves handling difficult thoughts and feelings. Expressing these difficult thoughts and feelings in group setting and through therapeutic writing reduces suffering. Engaging actively in CBT programs offers new viewpoints, aiding in the transition towards adaptation to the chronic pain situation. | 8 weeks. | Digitally recorded open and in-depth interviews. | 9/10. |
| Lou et al., 2022, Australia. | Qualitative research, descriptive study. | N = 6.  Sex: female 50.0%, male 33.33%, and non-binary 16.66%.  Average age: NR.  Age range: 21–71 years. | Pain diagnoses: FM and various chronic pain conditions, such as generalized pain, post-traumatic pain, and connective tissue disorders (timing and formality of diagnosis: NR). Duration of pain: > 3 months. | NR. | VATD (participants expressed and documented their pain experiences, mood changes, activities, and other relevant information through both visual art and written entries).  Diaries were written at home. | Control (N/A). | Throughout the 5-week period participants were encouraged to record as many diary entries as they desired. Intensity: NR. | Three main themes: Participants detailed their pain experiences, highlighting emotional and physical aspects.  Participants showed a strong motivation to grow and adapt despite their pain.  Daily activities and decisions were influenced by personal values and perceptions. | 5 weeks. | Self-reported pain diaries. | 8/10. |

**TableS2** summarizes key characteristics of the 24 included studies on therapeutic writing interventions for chronic pain. It includes study design, participant demographics, pain diagnosis and duration, psychological symptoms, intervention details, comparison conditions, writing task parameters, outcomes, assessment time points, measurement tools, and quality appraisal scores.

**Abbreviations: ACT** = Acceptance and Commitment Therapy, **ACTW** = ACT-based Writing, **EVID** = Educational Video, **ITW** = Integrative Therapeutic Writing, **NW** = Narrative Writing, **NWRIT** = Neutral Writing, **WED** = Written Emotional Disclosure, **VWED** = Variation of Written Emotional Disclosure, **WEM** = Written Enhanced Meaning, **TMW** = Time Management Writing, **VATD** = Visual Arts Diary, **WBF** = Written Benefit Finding, **WAE** = Written Anger Expression, **SWI** = Smartphone Writing Intervention

**Abbreviations of Diseases:** **CPP** = Chronic pelvic pain, **FM** = Fibromyalgia, **RA** = Rheumatoid arthritis, **SLE** = Systemic lupus erythematosus

**Surveys and Questionnaires**

**AEQ** = the Ambivalence Over Emotional Expression Questionnaire, **AIMS2** = the Arthritis Impact Measurement Scale, **BPI** = the Brief Pain Inventory, **CAB-V** = the Constructive Anger Behavior-Verbal Style Scale, **CAS** = the Clinical Anger Scale, **CPAQ** = the Chronic Pain Acceptance Questionnaire, **CPAQ-8** = the Chronic Pain Acceptance Questionnaire-8, **CPSS** = the Chronic Pain Self-Efficacy Scale, **CRP** = C-reactive protein, **CSQ** = The Coping Strategies Questionnaire, **CPVI** = the Chronic Pain Values Inventory, **CES-D** = the Center for Epidemiological Studies Depression Scale, **EAC** = the Emotional Approach Coping Scale, **ELS** = the Engaged Living Scale, **ESR** = the Erythrocyte Sedimentation Rate, **GHQ-12** = the General Health Questionnaire, **FFMQ-SF** = the Five Facet Mindfulness Questionnaire-Short Form, **HADS** = the Hospital Anxiety Depression Scale, **HMSE** = the Headache Management Self-Efficacy Scale, **HDI** = the Henry Ford Hospital Headache Disability Inventory, IIRS = the Illness Intrusiveness Rating Scale, **FSS** = Fatigue Severity Scale, **QOL** = the Quality of Life Scale, **LOT-R** = the Revised Life Orientation Test, **SACL** = the Stress and Arousal Checklist, **SPI** = the Sickness Impact Profile, **STAI-S** = the State-Trait Anxiety Scale**, BDI-II** = the Beck Depression Inventory-II, **BDI-SF** = the Beck Depression Inventory-Short Form, **MHAQ** = the Stanford modified Health Assessment Questionnaire, **MHC-SF** = the Mental Health Continuum-Short Form, **MIDAS** = the Migraine Disability Assessment Scale, MPI = the Multidimensional Pain Inventory, **MPQ** = the McGill Pain Questionnaire, **MOS** = the Medical Outcome Survey, **FIQ** = the Fibromyalgia Impact Questionnaire, **DAS** = the Disease Activity Rating Scale, **PANAS** = the Positive and Negative Affect Schedule, **PANAS-X** = the Positive and Negative Affect Schedule-Expanded Version, **PCS** = The Pain Catastrophizing Scale, **PDI** = the Pain Disability Index, **PGA** = the Physician’s Global Rating of Disease Activity, **PIPS** = the Psychological Inflexibility in Pain Scale, **POMS** = the Vigor subscale of the Profile of Mood States, **POMS-SF** = the Short Form of Profile of Mood State, **SCL-90-R** = the Symptom Checklist, **SCS** = the Self Compassion Scale, **SECS** = the Self Expression and Control Scale, **SF-8** = the Short-Form Health Survey, **SF-MPQ** = the Short-Form McGill Pain Questionnaire, **SF-36v2** = the SF-36v2 Health Survey, **SQS** = the Sleep Quality Scale, **SOPA** = the Survey of Pain Attitudes, **STAI** (Form Y-2) = the State-Trait Anxiety Inventory, **STAXI** = the State-Trait Anger Expression Inventory, **SWLS** = the Satisfaction with Life Scale, **VAS** = the Visual Analogue Scale.

**TableS3. Therapeutic Writing Paradigms and Techniques**

| **Writing Paradigm** | **Technique** | **Brief Description** | **Studies** |
| --- | --- | --- | --- |
| **Expressive Writing** | WED — Standard Written Emotional Disclosure | Writing about traumatic or stressful experiences | Broderick et al., 2004; Lumley et al., 2011b; Wetherell et al., 2005; Byrne-Davis et al., 2006; Smyth et al., 1999 |
|  | VWED — Emotional Expression and Reappraisal | Writing on stress and cognitive reappraisal | Broderick et al., 2005; Junghaenel et al., 2008 |
|  | VWED — Rheumatic Disease Focus | Writing about illness experiences | Danoff-Burg et al., 2006 |
|  | VWED — Ongoing Stress and Coping | Writing on persistent pain and coping | Gillis et al., 2006 |
|  | VWED — Chronic Pelvic Pain | Writing on negative emotions in pelvic pain | Norman et al., 2004 |
|  | VWED — Guided Emotional Expression, Meaning-Making, and Coping | Identification of a stressful or traumatic event for emotional exploration, meaning-making, and coping strategies. | Lumley et al., 2014 |
|  | VWED — Emotion-focused | Writing with structured prompts about pain-related and emotional experiences | Trompetter et a., 2015 |
|  | WAE — Directed Anger Expression | Writing anger toward a person or object | Graham et al., 2008 |
|  | WAE — Reflective Anger Diary | Daily reflection on anger experiences | Van Middendorp et al., 2010 |
| **Positive Writing** | WEM — Enhanced Meaning in Trauma | Writing to find meaning in trauma | Broderick et al., 2004 |
|  | WBF — Benefit-Focused Writing | Writing about positive aspects of illness | Danoff-Burg et al., 2006 |
|  | Self-Compassion Writing | Writing with self-kindness and emotional acceptance | Ziemer et al., 2015 |
|  | Self-Efficacy Writing | Writing to boost confidence in managing pain | Ziemer et al., 2015 |
| **Journal Writing** | SWI — Smartphone Diary with Therapist Feedback | CBT-based diary with therapist input | Kristjánsdóttir et al., 2013 |
|  | Electronic and Paper Diaries | Daily entries on pain and mood | Marceau et al., 2007 |
| **ACT-Based Writing** | ACTW — ACT-Based Writing | Writing based on ACT principles | Veillette et al., 2019 |
| **Integrative Writing** | ITW — Integrative Therapeutic Writing | Writing on pain, coping, and self-concept | Furnes and Dysvik, 2012; Furnes et al., 2014; Furnes et al., 2015 |
| **Narrative Writing** | PV — Photovoice | Photos and narratives of pain experience | Baker & Wang, 2006 |
|  | VATD — Visual Art Diary | Combined art and writing narratives | Lou et al., 2022 |
|  | NW — (Free-form) Narrative Writing | Unstructured writing on pain and coping | Brown et al., 2010 |

**TableS3** summarizes 20 distinct writing techniques categorized under six therapeutic writing paradigms. Each technique is briefly described and linked to the studies in which it was applied. Techniques sharing the same abbreviation (e.g., VWED, WAE) are differentiated by their specific focus or implementation. Full methodological and contextual descriptions are provided in **tableS2**.

**TableS4. CERQual Summary of Findings: Experienced Health Effects**

| **Theme (synthesized finding)** | **Contributing studies** | **Methodological limitations** | **Coherence** | **Adequacy of data** | **Relevance** | **Confidence level** |
| --- | --- | --- | --- | --- | --- | --- |
| Making the Invisible Visible | Baker et al. (2006); Lou et al. (2022); Brown et al. (2010) | Moderate: reflexivity and ethics gaps in Baker and Lou | High: consistent across modalities (photovoice, visual diaries, narrative writing) | Moderate: rich examples, limited number of studies | High: directly relevant to chronic pain expression | Moderate confidence |
| Emotional Catharsis, Cognitive Reappraisal, and Meaning-Making | Byrne-Davis et al. (2006); Brown et al. (2010); Lou et al. (2022); Baker et al. (2006); Furnes and Dysvik, 2012; Furnes et al. (2014, 2015) | Minor to moderate: Byrne-Davis and Baker scored lower | High: strong thematic convergence across diverse modalities | High: rich data from seven studies | High: directly relevant to emotional processing in chronic pain | High confidence |
| Enhanced Self-Awareness and Identity Reconstruction | Brown et al. (2010); Furnes et al. (2015); Lou et al. (2022) | Minor: all studies scored 8–9/10 | High: clear narrative of identity work and introspection | Moderate: fewer studies, but rich participant quotes | High: relevant to psychological adaptation | Moderate confidence |
| Connection, Validation, and Shared Understanding | Furnes et al. (2014); Baker et al. (2006) | Moderate: ethical gaps in Baker | High: strong coherence in group-based interventions | Moderate: limited number of studies, vivid examples | High: relevant to social dimensions of pain | Moderate confidence |
| Therapeutic Empowerment and Self-Management | Furnes and Dysvik (2012); Furnes et al. (2014, 2015); Brown et al. (2010) | Minor: all studies scored 8–10/10 | High: consistent findings across CBT and narrative writing | High: strong data supporting self-efficacy and coping | High: directly relevant to chronic pain management | High confidence |
| Challenges and Considerations | Byrne-Davis et al. (2006); Furnes et al. (2012, 2015); Baker et al. (2006) | Moderate: lower scores in Byrne-Davis and Baker | High: coherent across studies and supported by quantitative findings | High: rich descriptions of emotional strain and attrition | High: highly relevant to intervention design | Moderate confidence |

**TableS4** presents a CERQual summary of the six synthesized findings derived from seven qualitative studies exploring therapeutic writing interventions in adults with chronic pain. Confidence levels ranged from moderate to high across all themes. Findings related to *Emotional Catharsis, Cognitive Reappraisal, and Meaning-Making* and *Therapeutic Empowerment and Self-Management* were supported by high-quality studies with rich, coherent data, resulting in high confidence ratings. Themes such as *Making the Invisible Visible*, *Identity Reconstruction*, and *Shared Understanding* were rated with moderate confidence due to minor methodological limitations and fewer contributing studies. The theme *Challenges and Considerations* also received moderate confidence, reflecting consistent but emotionally complex participant experiences and limitations in study design. Overall, the synthesis highlights the psychological and relational benefits of therapeutic writing, while emphasizing the need for tailored facilitation and ethical sensitivity in intervention delivery.

**TableS5. Summary of Findings: Pain Management and Reduction (GRADE and CERQual)**

| **Outcome** | **Studies Contributing** | **Risk of bias** | **Inconsistency** | **Indirectness** | **Imprecision** | **Publication bias** | **Certainty of evidence** |
| --- | --- | --- | --- | --- | --- | --- | --- |
| Pain reduction through expressive writing | Lumley et al. (2011b); Broderick et al. (2005); Junghaenel et al. (2008); Norman et al. (2004); Graham et al. (2008); Van Middendorp et al. (2010); Trompetter et al. (2015) | Moderate: several studies had unclear randomization, blinding, and incomplete follow-up | Moderate: effects varied across conditions and time points | Low: interventions and populations were relevant | Moderate: small sample sizes and short follow-up durations | Possible: limited number of high-quality trials | **Low to moderate certainty** |
| Pain reduction through positive writing | Danoff-Burg et al. (2006); Ziemer et al. (2015) | Serious: Both studies had multiple methodological limitations. Danoff-Burg scored 8/13 (unclear blinding, incomplete follow-up). Ziemer scored 4/13 with major issues in randomization and baseline comparability. | Not Serious: No major contradictions in direction of effect between studies. | Low: Interventions and populations were directly relevant to the clinical question. | Serious: Small sample sizes and lack of power calculations reduce confidence in effect estimates. | Possible: Only two studies with low methodological quality; selective reporting cannot be ruled out. | **Low certainty** |
| Pain reduction through ACT-based writing | Veillette et al. (2019) | Serious: Only 6/12 JBI criteria met. Key issues include lack of blinding (participants, providers, assessors), unclear follow-up, and allocation concealment. | Not Applicable: Only one study, so inconsistency cannot be assessed. | Low: Intervention (ACT-based writing) and population were relevant to the clinical question. | Serious: Single study with small sample size and no reported power analysis limits confidence in effect estimates. | Possible: Single study with moderate quality; risk of selective reporting cannot be ruled out. | **Low certainty** |
| Pain reduction via digital and group-based formats (hybrid GRADE + CERQual) | Kristjánsdóttir et al. (2013); Marceau et al. (2007); Furnes and Dysvik (2012); Furnes et al. (2014, 2015); Brown et al. (2010); Lou et al. (2022) | GRADE: moderate risk of bias, inconsistent results, limited follow-up  CERQual: minor limitations, strong coherence, high relevance | Mixed: modest clinical effects but consistent psychosocial benefits | Mixed: relevant but hard to isolate writing’s contribution | Mixed: small samples, qualitative richness, limited RCTs | Likely: few controlled trials, qualitative publication bias possible | **Moderate certainty** |

**TableS5** summarizes the certainty of evidence for therapeutic writing interventions targeting pain management, based on GRADE and CERQual assessments. Expressive and positive writing interventions showed modest effects on pain reduction, though methodological limitations and small sample sizes lowered the certainty of evidence. ACT-based writing was supported by a single study with moderate quality, resulting in low certainty. For digital and group-based formats, a hybrid assessment was applied: quantitative studies provided limited support for clinical pain reduction, while qualitative studies consistently highlighted psychosocial benefits such as emotional insight, peer validation, and enhanced engagement. The overall confidence rating for this outcome reflects the integration of both quantitative and qualitative findings.

**TableS6. Summary of Findings: Mental Health Outcomes (GRADE and CERQual)**

| **Outcome** | **Studies Contributing** | **Risk of Bias** | **Inconsistency** | **Indirectness** | **Imprecision** | **Publication Bias** | **Certainty of Evidence** |
| --- | --- | --- | --- | --- | --- | --- | --- |
| Mood and affect (short-term) | Wetherell et al. (2005); Broderick et al. (2004) | Moderate: small samples, limited blinding | Moderate: transient effects | Low: interventions relevant | Moderate: short-term follow-up | Possible: few trials | Low to moderate certainty |
| Psychological well-being and emotional activation | Danoff-Burg et al. (2006); Gillis et al. (2006); Broderick et al. (2005); Junghaenel et al. (2008); Trompetter et al. (2015); Norman et al. (2004) | Moderate: varied designs and reporting | High: effects moderated by traits | Low: relevant interventions | Moderate: small samples | Possible: selective reporting | Low certainty |
| Depression, anxiety, psychological flexibility | Trompetter et al. (2015); Veillette et al. (2019) | Moderate: lack of blinding | Low: consistent improvements | Low: directly relevant | Moderate: sustained effects, small samples | Possible: few trials | Moderate certainty |
| Emotional resilience and psychological adaptation | Furnes and Dysvik (2012); Furnes et al. (2014, 2015) | Low: strong qualitative rigor | Low: coherent findings | Low: highly relevant | Moderate: qualitative depth, small samples | Possible: qualitative publication bias | High confidence |
| Emotional insight and meaning-making | Brown et al. (2010); Baker and Wang (2006); Lou et al. (2022) | Moderate: qualitative limitations | Moderate: varied personal reflections | Low: relevant formats | Moderate: limited sample depth | Possible: narrative bias | Moderate confidence |
| Perceived psychological benefit | Byrne-Davis et al. (2006) | Moderate: subjective reporting | Moderate: catharsis varies | Low: relevant experience | High: small sample, limited depth | Possible: self-report bias | Low to moderate confidence |
| Depressed mood and emotional regulation | Graham et al. (2008); van Middendorp et al. (2010) | Moderate: emotion regulation not controlled | Moderate: moderated by style | Low: relevant interventions | Moderate: small samples | Possible: limited replication | Low to moderate certainty |
| Positive affect and life satisfaction | Danoff-Burg et al. (2006); Ziemer et al. (2015); Lou et al. (2022) | Moderate: mixed methods | Low: consistent subjective gains | Low: relevant populations | Moderate: modest clinical effects | Possible: few trials | Moderate overall confidence |
| Mental health outcomes (combined) | Trompetter et al. (2015); Veillette et al. (2019); Furnes et al. (2014, 2015) | Moderate: mixed-methods rigor | Low: consistent across domains | Low: interventions relevant | Moderate: small samples, sustained effects | Possible: limited trial pool | Moderate to high overall confidence |

**TableS6** presents a GRADE-style summary of mental health outcomes associated with therapeutic writing interventions. Quantitative studies were assessed using the GRADE framework, while qualitative and mixed-methods findings were mapped to equivalent domains (e.g., coherence to consistency, relevance to indirectness) to ensure comparability. Expressive writing, positive writing, and ACT-based writing showed improvements across mental health domains. Integrative and narrative writing approaches supported psychological adaptation and emotional insight, while emotion-focused and benefit-based writing yielded modest clinical effects alongside meaningful subjective gains.

**TableS7. GRADE Summary of Findings: Physical Health Outcomes**

| \| **Outcome** \| **Studies Contributing** \| **Risk of Bias** \| **Inconsistency** \| **Indirectness** \| **Imprecision** \| **Publication Bias** \| **Certainty of Evidence** \| \| --- \| --- \| --- \| --- \| --- \| --- \| --- \| --- \| \| Reduction in disease activity (RA) \| Lumley et al. (2014); Smyth et al. (1999) \| Low: blinded assessors, good randomization \| Low: consistent effects across trials \| Low: direct RA samples and outcomes \| Moderate: modest sample sizes \| Possible: few studies \| Moderate certainty \| \| Fatigue reduction (short-term) \| Danoff-Burg et al. (2006); Broderick et al. (2005); Junghaenel et al. (2008) \| Moderate: self-report only, limited blinding \| Moderate: short-lived effects \| Low: interventions relevant \| Moderate: no long-term follow-up \| Possible: few trials \| Low certainty \| \| Physical disability (expressive writing) \| Danoff-Burg et al. (2006); Gillis et al. (2006); Norman et al. (2004); Lumley et al. (2014) \| Moderate: subgroup variation, unclear blinding \| High: inconsistent effects across groups \| Low: relevant interventions \| Moderate: small samples \| Possible: selective reporting \| Low to moderate certainty \| \| Pain-related disability (ACT-based writing or VWED) \| Trompetter et al. (2015); Veillette et al. (2019) \| Moderate: lack of blinding and allocation concealment \| Low: consistent across studies \| Low: directly relevant interventions \| Moderate: sustained effects, small samples \| Possible: few trials \| Moderate certainty \| |
| --- | --- | --- | --- | --- | --- | --- | --- | --- | --- | --- | --- | --- | --- | --- | --- | --- | --- | --- | --- | --- | --- | --- | --- | --- | --- | --- | --- | --- | --- | --- | --- | --- | --- | --- | --- | --- | --- | --- | --- | --- |

**TableS7** presents a GRADE-style summary of physical health outcomes associated with therapeutic writing interventions. Quantitative studies were evaluated using the GRADE framework. Fatigue-related improvements were observed in VWED and benefit-focused writing, though effects were short-lived and limited to self-report measures. Expressive writing showed small to moderate effects on physical disability in some populations, with subgroup variation based on emotional coping styles. Moderate-quality trials support the finding that both an ACT-based writing intervention and a VWED intervention led to more consistent and sustained reductions in pain-related disability. Overall, physical health outcomes were modest and context-dependent, with durability and objective assessment remaining key limitations.
